# Combined In Silico and In Vitro Analyses to Assess the Anticancer Potential of Thiazolidinedione–Thiosemicarbazone Hybrid Molecules

**DOI:** 10.3390/ijms242417521

**Published:** 2023-12-15

**Authors:** Agata Paneth, Barbara Kaproń, Tomasz Plech, Roman Paduch, Nazar Trotsko, Piotr Paneth

**Affiliations:** 1 Chair and Department of Organic Chemistry, Faculty of Pharmacy, Medical University of Lublin, 20-059 Lublin, Poland; agata.paneth@umlub.pl; 2 Department of Clinical Genetics, Faculty of Medicine, Medical University of Lublin, 20-080 Lublin, Poland; 3 Department of Pharmacology, Faculty of Health Sciences, Medical University of Lublin, 20-080 Lublin, Poland; tomasz.plech@umlub.pl; 4 Department of Virology and Immunology, Institute of Biological Sciences, Faculty of Biology and Biotechnology, Maria Curie-Skłodowska University, 20-033 Lublin, Poland; roman.paduch@mail.umcs.pl; 5 Institute of Applied Radiation Chemistry, Faculty of Chemistry, Lodz University of Technology, 90-924 Lodz, Poland

**Keywords:** thiazolidinedione, thiosemicarbazone, molecular docking, HDACs, PPARγ, cytotoxic effect, apoptosis, cell migration, hemolytic activity

## Abstract

The number of people affected by cancer and antibiotic-resistant bacterial infections has increased, such that both diseases are already seen as current and future leading causes of death globally. To address this issue, based on a combined in silico and in vitro approach, we explored the anticancer potential of known antibacterials with a thiazolidinedione–thiosemicarbazone (TZD–TSC) core structure. A cytotoxicity assessment showed encouraging results for compounds **2**–**4**, with IC_50_ values against T98G and HepG2 cells in the low micromolar range. TZD–TSC **3** proved to be most toxic to cancer cell lines, with IC_50_ values of 2.97 ± 0.39 µM against human hepatoma HepG2 cells and IC_50_ values of 28.34 ± 2.21 µM against human glioblastoma T98G cells. Additionally, compound **3** induced apoptosis and showed no specific hemolytic activity. Furthermore, treatment using **3** on cancer cell lines alters these cells’ morphology and further suppresses migratory activity. Molecular docking, in turn, suggests that **3** would have the capacity to simultaneously target HDACs and PPARγ, by the activation of PPARγ and the inhibition of both HDAC4 and HDAC8. Thus, the promising preliminary results obtained with TZD–TSC **3** represent an encouraging starting point for the rational design of novel chemotherapeutics with dual antibacterial and anticancer activities.

## 1. Introduction

According to the latest estimates, the number of people affected by cancer and antibiotic-resistant bacterial infections has increased, such that both diseases are already seen as current and future leading causes of death globally [1,2]. Something especially alarming is the global emergence and rapid spread of multi- and pan-resistant bacteria that cause infections which are not treatable with the available treatment options [3]. Numerous antibiotic resistance mechanisms, with special attention paid to antibiotic resistance genes, have been identified so far, and the most serious are those providing resistance to the few reserve antibiotics available in the clinic for targeted use in multidrug-resistant infections [4,5,6]. According to the Final Report and Recommendations on Tackling Drug-Resistant Infections Globally, it is estimated that, without intervention, global deaths associated with antimicrobial resistance could reach 10 million annually by 2050 [7]. The economic impact of antimicrobial resistance on healthcare facilities and systems is also significant; in addition to death and long-term disability, prolonged illness results in longer hospital stays, the need for more expensive and intensive care, and financial challenges for those affected [3]. Moreover, infectious agents and chronic infections are one of the main causes of cancer and/or associated risk factors, due to the induction of chronic inflammation and the chronic suppression of the immune system. The induction of chronic inflammation, as a result of a continued immune response to a continuous persistent infection by a pathogen, together with the chronic suppression of the immune system by the infectious agent, leads to chronic cell proliferation and a greater risk of oncogenic transformation [8]. Likewise, anticancer therapy itself and cancer-related physical debility often pave the way for a variety of opportunistic infections. Consequently, the development of novel antimicrobial agents as functional therapeutics to fight against infectious diseases and cancer is urgently needed.

The development of chemoresistance in cancer cells against mono-targeting anticancer agents, via the modulation of multiple survival pathways, along with target non-specificity due to tumor heterogeneity, are the major causes of failure of cancer therapy. In overcoming these concerns, a multi-targeting approach with reduced detrimental effects, such as drug–drug interactions, side effects, and poor patient compliance, may have profoundly high potential in this respect [9,10,11,12,13,14]. In the complex and dynamic process of tumorigenesis, epigenetics—through DNA methylation, histone modification, chromatin remodeling, and noncoding RNA regulation—contribute significantly [15]. Tumor cell activation is effectively regulated by the downregulation of tumor-associated antigens and the loss of antigen processing and presentation machinery, as well as the expression of a tumor-promoting balance in co-stimulatory and co-inhibitory molecules, which facilitate an escape from chemotherapy [16,17,18]. Given the importance of epigenetic regulation in cancers, the recent focus of anticancer drug discovery has been directed to epigenetic targets [19,20,21]. There are numerous regulatory enzymes involved in epigenetic modification [22]. Among them, histone deacetylases (HDACs) are critically involved in physiological processes such as gene transcription and cellular homeostasis [23,24]. Recent studies have shown that the dysregulation of these enzymes, caused by changes in the protein’s amino acid sequence, is closely correlated with tumor onset and progression [22]. There are various HDAC inhibitors that have shown promising anti-cancer efficacy in preclinical and clinical trials. For example, suberoylanilide hydroxamic acid has been utilized in clinical treatments for cutaneous T-cell lymphoma [25], belinostat has been approved for patients with peripheral T-cell lymphoma [26], and panobinostat is the first HDAC inhibitor approved for patients with relapsed multiple myeloma [27].

Peroxisome proliferator-activated receptors (PPARs), in turn, are ligand-activated transcription factors that control the expression of a large number of genes involved directly or indirectly in glucose homeostasis, adipogenesis, and inflammation [28]. PPARs include three subtypes: PPARα, PPARδ, and PPARγ [29]. The subtype PPARγ is the most widely studied and its activation increases glucose uptake and utilization, stimulates fatty acid storage, enhances insulin signaling, and decreases gluconeogenesis, thereby improving insulin sensitivity [30]. PPARγ is the receptor for well-known thiazolidinedione (TZD)-based full agonists (e.g., pioglitazone and rosiglitazone), which belong to the antidiabetic drugs for the treatment of type 2 diabetes mellitus [31]. In addition, to the role of PPARγ in macronutrient metabolism, it is also vital to cancer cell growth regulation. Several antineoplastic effects, such as the induction of apoptosis and differentiation, have been observed for PPARγ agonists, both in vitro and in vivo, as well as in clinical trials [32,33,34,35,36,37,38,39,40,41]. Due to the undesirable side effects associated with full agonist activity, PPARγ partial agonists (e.g., balaglitazone and netoglitazone), with a reduced incidence of such effects in preclinical models, have been discovered [42,43].

**Figure 1:**
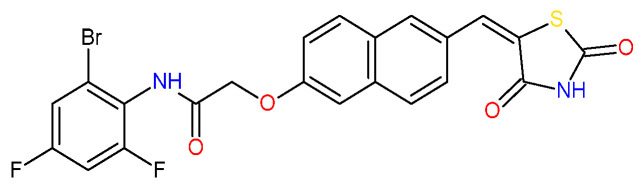
TZD-based compound **1** with dual-targeting PPARγ and HDAC4 ability and anticancer potency [24].

Recent reports suggest that the synergistic/additive effects of PPARγ agonists and HDAC inhibitors increase their cytotoxic effects against tumor cells, resulting in proliferation arrest and apoptosis induction. In some cancers, combining low doses of a PPARγ ligand with a weak HDAC inhibitor proved to be more successful than treatments with either drug alone [24]. On the basis of these results, a series of novel TZD-based compounds, simultaneously targeting PPARγ and HDACs, with in vitro and in vivo antitumor effects, were discovered by Ramaa and co-workers [24]. Such an example of a dual targeting agent is compound **1**, with activity toward PPARγ and HDAC4 at a low micromolar concentration (Figure 1). This compound was also found to exhibit antiproliferative effects against CEM cells with an IC_50_ value of 9.6 μM. Furthermore, it induced apoptosis and caused a significant DNA fragmentation in cell cycle analysis. Collectively, these data suggest that TZD-based compounds, with dual targeting ability via the partial activation of PPARγ and the selective inhibition of HDAC, may become novel therapeutic options for anticancer therapy.

Recently, we reported the development of thiazolidinedione–thiosemicarbazones (TZD–TSCs) as novel antibacterials [44]. Some of these compounds exhibited strong in vitro potency with minimal inhibitory concentrations (MICs) in the range of 8.48–16.94 µM, as illustrated by **2** and **3** (for chemical structures see table in Section 2.2). An investigation of the toxicity profile of closely related hybrid molecules [45] revealed their acceptable toxicity and their lack of genotoxic activity and hemolytic effects. Following the promising data presented by Ramaa and co-workers [24], in this study we proposed that the biological potency of TZD–TSCs, especially as anticancer compounds, could be further expanded. Here, we present the results of a combined in silico and in vitro approach that allowed us to conclude that the investigated structures have potential for multi-target drug design and development.

## 2. Results and Discussion

### 2.1. Rationale

Although cancer and infectious diseases are totally distinct categories of ailments, the potential link between cancer and infection has been addressed in prior research. In this context, the most firmly established relationships exist between carcinogenesis and oncogenic infectious pathogens, between cancer-associated bacteria and the effectiveness of cancer treatment, and between cancer therapy and the development of an opportunistic infection that may be fatal, especially in patients with immune suppression [46,47,48]. According to an analysis of the global incidence of the burden of cancers attributable to infections in 2018, approximately 2.2 million new cancer diagnoses were caused by infectious agents worldwide [49]. Furthermore, autopsy studies demonstrate that approximately 60% of deaths in cancer patients, especially those with underlying hematological malignancies, are infection-related. Additionally, despite the fact that there are less data on infectious mortality in patients with solid organ tumors, it is estimated that in approximately 50% of these patients, infection is either the primary or an associated cause of death [50]. Finally, intracellular pathogens, including bacteria, contribute to anticancer drug resistance. Bacteria can metabolize chemotherapeutics and alter autophagy in cancer cells, leading to enhanced drug resistance. Additionally, certain Gram-positive bacteria can migrate to distant sites, along with primary tumor cells, promoting cancer growth and metastasis [48]. Thus, the simultaneous suppression of pathogenic microorganisms during anticancer drug therapy can not only interrupt or completely suppress the tumor growth, but also ultimately protect patients with cancer from infection [51]. Hence, the development of dual-acting antibacterial and anticancer therapeutics, which would affect the malignant process and simultaneously decrease the risk of patients’ deaths due to infection, remains an attractive goal in the contemporary medicinal chemistry and drug discovery arena [48,51,52]. Examples of such dual-acting therapeutics include interferons, endostatin, epidermal growth factor receptor (EGFR) antagonists [46], and antitumor antibiotics [53]. With regard to antitumor antibiotics, their specific medical application for cancer treatment is well established in clinical cancer care. According to research findings, these drugs can promote cancer apoptosis, inhibit cancer growth and prevent cancer metastasis. For these reasons, antitumor antibiotics are amongst the most important of the cancer chemotherapeutic agents [53]. Among novel chemical entities in preclinical studies, in turn, the potential role of TZD-based compounds for future dual antimicrobial and anticancer applications has already been pointed out by other researchers [53,54,55,56,57].

### 2.2. Molecular Docking

**Figure 2:**
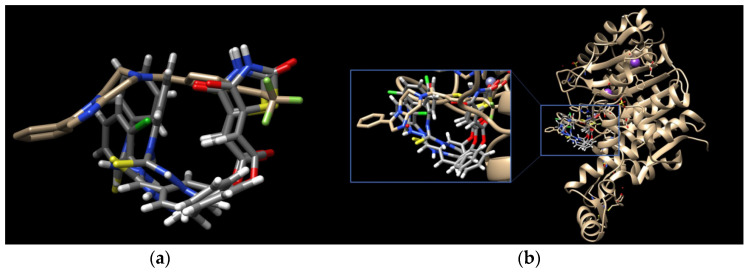
(**a**) Docked conformations of TZD–TSCs **2**–**4** superimposed with the crystal conformation of TFG inhibitor (light brown) in complex with HDAC4_o_ (PDB code: 2VQJ); (**b**) binding mode of TZD–TSCs **2**–**4** into HDAC4_o_. Nitrogen atoms are blue, oxygen atoms are red, and sulfur atoms are yellow.

**Figure 3:**
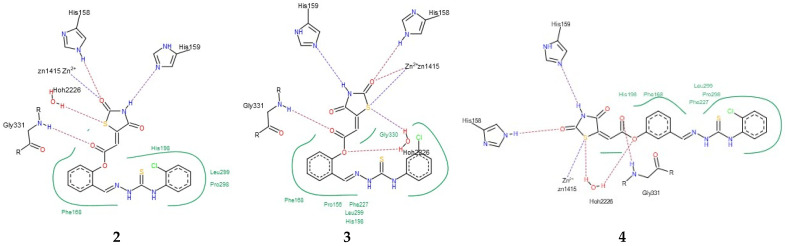
PoseView images for TZD–TSCs **2**–**4** into the open conformation of HDAC4_o_ (PDB code: 2VQJ). Hydrophobic interactions are displayed as green contact curves; H-bonds and metal interactions are presented as dashed lines.

Having in view the pioneering findings presented by Ramaa and co-workers [24], our studies were conducted, starting with a docking simulation, to explore the potential impacts of HDAC4, HDAC8, and PPARγ ligand binding domain (LBD) on the binding of TZD–TSCs. For these studies, crystal structures deposited in Protein Data Bank, which were previously utilized in the docking of TZDs [24], were selected. Thus, for the docking of TZD–TSCs **2**–**5** with HDAC4, both open and closed conformations of HDAC4 were utilized, using crystal structures under PDB code 2VQJ [58] and 4CBY [59], respectively. As presented in Table 1, except for ligand **5** which was not recognized by both open and closed HDAC4, docking to the open conformation of HDAC4 yielded more favorable binding scores, compared to closed. Consequently, the binding modes of TZD–TSCs **2**–**4** into the large binding groove of HDAC4 have been analyzed in detail. As presented in Figure 2, although ligands **2**–**4** bind to the active site in a U-shaped conformation, their docking scores remain comparable to that of the native inhibitor TFG (2,2,2-trifluoro-1-{5-[(3-phenyl-5,6-dihydroimidazo [1,2-a]pyrazin-7(8H)-yl)carbonyl]thiophen-2-yl}ethane-1,1-diol). Calculations suggest that, in analogy to previously reported TZDs [24], TZD–TSCs **2**–**4** bind the catalytic zinc ion through the TZD head group (Figure 3). Among them, the docking of **3** predicts that the ligand binds the catalytic zinc ion in a bidentate fashion, while the others chelate the catalytic zinc ion in a monodentate manner. It is noteworthy that these binding poses were highly superimposable with a TFG native inhibitor in a complex with HDAC4. In this the sulphur– or the oxygen–Zn2+ coordination pose, the oxygen atoms of TZD head group of ligands 2-4 interact through H-bonds with His158 while the nitrogen atoms serve as hydrogen donors for His159. For the TZD head groups of **2** and **4**, H-bond interactions between the sulfur atom and HOH2226 are also predicted. These binding modes of ligands **2**–**4** are further stabilized by the H-bond contact between the carbonyl oxygen atom and Gly331, as well as numerous hydrophobic interactions between the thiosemicarbazone tail, alternatively distal chlorophenyl group, and surrounding residues. Some side chains in protein which are involved in intermolecular interactions with ligands **2**–**4** (e.g., Pro156, His159, Phe168, and Gly330) are identical to those previously reported for TZDs [24].

**Figure 4:**
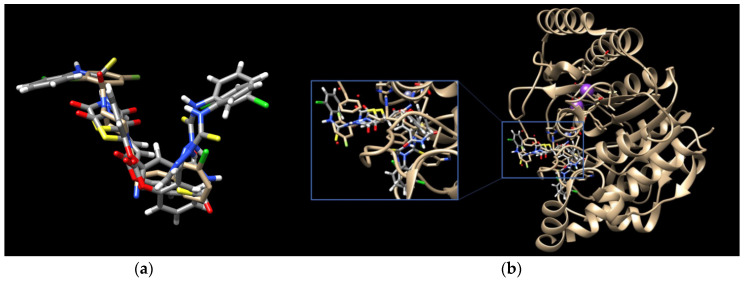
(**a**) Docked conformations of TZD–TSCs **2**–**4** superimposed with the crystal conformation of 0DI inhibitor (*light brown*) in complex with HDAC8 (PDB code: 3SFF); (**b**) binding modes of TZD–TSCs **2**–**4** into HDAC8. Nitrogen atoms are blue, oxygen atoms are red, and sulfur atoms are yellow.

**Figure 5:**
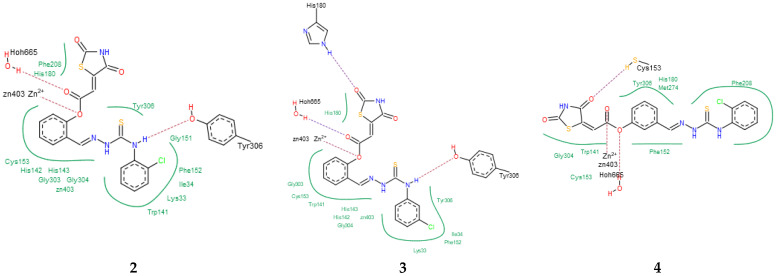
PoseView images for TZD–TSCs **2**–**4** into the active site of HDAC8 (PDB code: 3SFF). Hydrophobic interactions are displayed as green contact curves; H-bonds and metal interactions are presented as dashed lines.

To elucidate the binding modes of TZD–TSCs **2**–**5** at the HDAC8 active site, the crystal structure under PDB code 3SFF [60] was selected. In analogy with the results from docking to HDAC4_o_, the ligands bind to the active site in a U-shaped conformation (Figure 4), yielding docking scores comparable to the native inhibitor 0DI, except for **5**, for which no interactions with HDAC8 were recognized (Table 1). Contrary to the results from docking to HDAC4_o_, however, ligands **2**–**4** bind the catalytic site with their ester moiety instead of the TZD head group, chelating the zinc cation in a monodentate manner (Figure 5). In this regard, the calculations suggest that the metal ion can be alternatively coordinated through the oxygen atom of the carbonyl group, as for **4**, or the central phenoxy moiety, as for **2** and **3**. In addition to zinc complexation, the ester group of ligands ***2***–**4** is H-bonded to HOH665, while, for **2** and **3**, H-bond interactions between the NH group of the thiosemicarbazone tail and Tyr306 are also predicted. The binding modes of TZD–TSCs **2**–**4** are further stabilized by numerous hydrophobic interactions with side chains of residues from both the acetyl–lysine substrate tunnel (His143, Gly151, Phe152, His180, and Phe208) and the acetate product release channel (Ile34, Trp141, Gly303, Gly304, and Tyr306). These binding modes of ligand **2**–**4** were, as a result, highly superimposable with the native inhibitor 0DI in a complex with HDAC8. Collectively, these findings illustrate the potential of TZD–TSCs **2**–**4,** especially **2** and **3**, as dual HDAC4/8 inhibitors.

**Figure 6:**
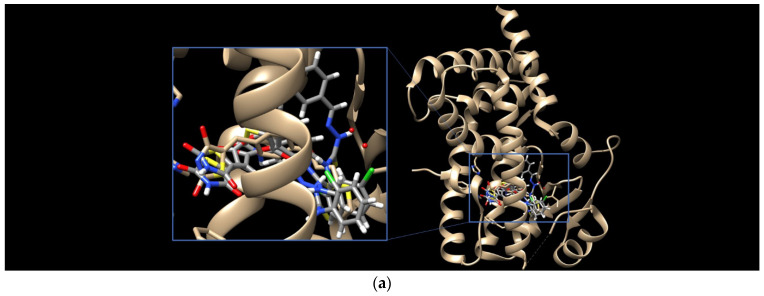

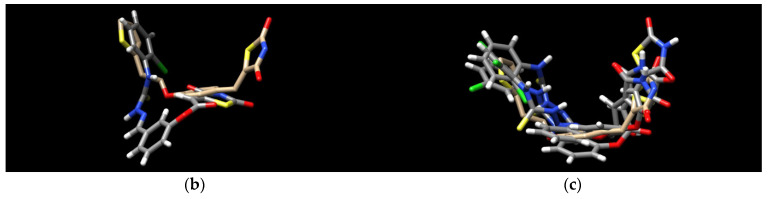
(**a**) Binding modes of TZD–TSCs **2**–**5** into the active site of PPARγ LBD (PDB code: 6DGR); the docked conformations of: (**b**) TZD–TSC **4**, and (**c**) TZD–TSCs **2**, **3**, and **5**, superimposed with the crystal conformation of native agonist GDY (*light brown*). Nitrogen atoms are blue, oxygen atoms are red, and sulfur atom is yellow.

**Figure 7:**
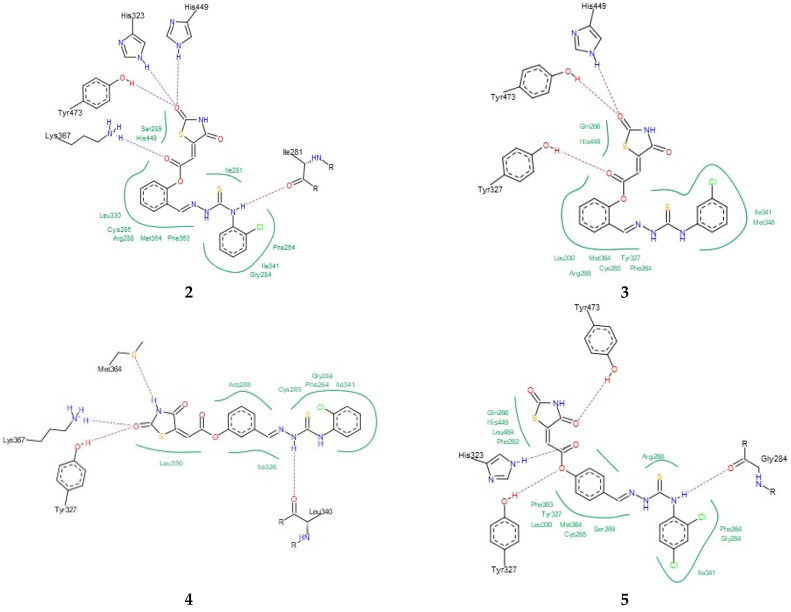
PoseView images for TZD–TSCs **2**–**5** into the active site of PPARγ LBD (PDB code 6DGR). Hydrophobic interactions are displayed as green contact curves; H-bonds are presented as dashed lines.

Finally, the binding modes of TZD–TSCs **2**–**5** to PPARγ LBD (PDB code 6DGR [61]) were investigated. As shown in Figure 6, all of them were identified as binding to PPARγ LBD in a U-shaped conformation, in analogy to the previously reported TZDs [24] and other known TZD-based agonists [61,62]. Additionally, all of them were identified as binding to the PPARγ LBD, with a binding mode very similar to the native agonist GDY ((5Z)-5-({4-[2-(thiophen-2-yl)ethoxy]phenyl}methylidene)-1,3-thiazolidine-2,4-dione) (Figure 6), and, at the same time, with much higher binding affinity (Table 1). As shown in Figure 7, the oxygen atoms of the TZD head groups of **2**, **3**, and **5** are H-bonded to the phenolic proton of Tyr473, and this interaction is essential for PPARγ activation [63]. In addition to the H-bond interaction with Tyr473, the TZD head groups of **2**, **3**, and **5** are stabilized by direct and indirect contact with the surrounding residues Phe282, Gln286, Ser289, His323, His449, and Leu469. The phenoxy group of ligands **2**, **3**, and **5**, in turn, is placed in the center of the LBD and makes hydrophobic interactions with Cys285, Tyr327, Leu330, Phe363, and Met364, while, for the ester group, H-bond contacts with His323, Tyr327, and Lys367 were also predicted. The thiosemicarbazone tail, together with the distal chlorophenyl group, lies in the subpocket between H3, the *β*-sheet, and the Ω-loop, establishing numerous intermolecular interactions with the surrounding residues. Most of these interactions, especially with Ile341 (β-sheet), Met348 (β-sheet), and Phe264 (Ω-loop), are identical to those predicted for the pioneering series of TZDs [24].

Interestingly, the docking of ligand **4**, for which the best potency towards the PPARγ LBD was predicted, revealed that its U-shape binding pose is slightly different to that of the other TZD–TSCs. As presented in Figure 6b, the superimposition of **4** on the cocrystal structure of the native agonist GDY revealed that its TZD head group undergoes a shift toward H3, thus preventing the formation of a key H-bond with the residue Tyr473. Instead of this, H-bond interactions between the TZD head group and Tyr327, Met364, and Lys367 have been predicted (Figure 7). The phenoxy–carbonyl group is also placed in the center of the LBD and makes several hydrophobic interactions with the surrounding residues. In this pose, the nitrogen atom of the thiosemicarbazone tail interacts, through a H-bond, with Leu340 (*β*-sheet), while, for the distal chlorophenyl group, several hydrophobic contacts with side chains from the *β*-sheet (Ile341) and *β*-sheet subpocket (Phe264) were predicted. Thus, based on docking simulations, we theorized that the hybrid compounds TZD–TSCs **2**–**5** would have the capacity to simultaneously target HDACs and PPARγ.

### 2.3. Chemistry

**Scheme 1:**
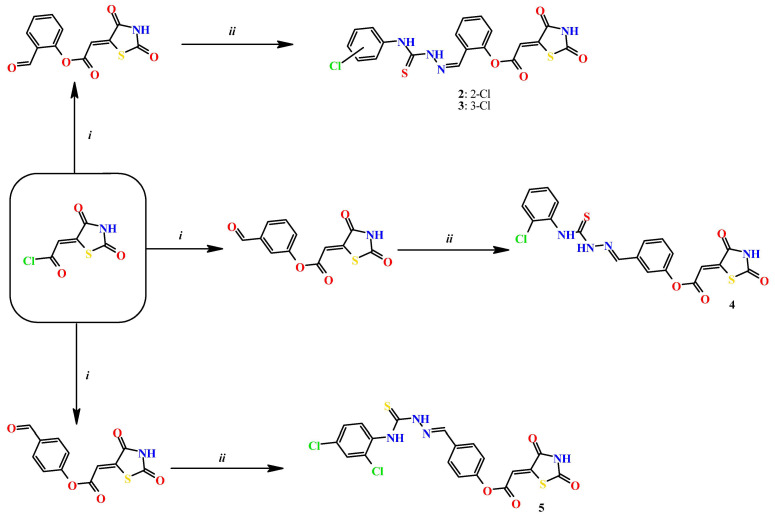
*Reagent and conditions:* (*i*) corresponding hydroxybenzaldehyde, pyridine, 1,4-dioxane, rt, 2h, acidified with dilute HCl; (*ii*) corresponding thiosemicarbazide, anhydrous ethanol, reflux.

Based on docking calculations, it was deducted that TZD–TSCs **2**–**5** are able to act as potential dual HDAC and PPARγ targeting agents; hence, they were considered for further evaluation, with regard to their cytotoxic potency. Before in vitro experiments, these hybrid molecules were screened for pan assay interference compounds (PAINS), using SwissADME web server (http://www.swissadme.ch) (accessed on 20 November 2023). Since no alerts for PAINS were detected for any of them, the compounds were resynthesized according to a two-step synthetic procedure, described in detail elsewhere [44]. Briefly, a condensation reaction of (2,4-dioxo-1,3-thiazolidin-5-ylidene)acetyl chloride with its corresponding hydroxybenzaldehydes resulted in formylphenyl (2,4-dioxo-1,3-thiazolidin-5-ylidene)acetates. A further reaction with the relevant 4-(chlorophenyl)thiosemicarbazides produced the final TZD-TSCs **2**–**5** (Scheme 1).

### 2.4. Cytotoxicity Assessment

The effects of TZD–TSCs **2**–**5** on the cellular viability of a panel of cell lines were investigated through an 3-(4,5-dimethylthiazol-2-yl)-2,5-diphenyl-2H-tetrazolium bromide (MTT) assay, following a 24 h treatment with the compounds. The panel consisted of three solid tumor cells: T98G (glioblastoma multiforme), HepG2 (hepatocellular carcinoma, epithelial), and HT29 (colon adenocarcinoma, epithelial), as well as noncancerous cells CCD 841 CoTr (normal colon, epithelial).

Primary single dose screenings of TZD–TSCs **2**–**5** were performed at a concentration of 200 μg/mL, and those that elicited a greater than 80% inhibition of cell proliferation were analyzed at several concentrations, to find their half-maximal inhibitory concentration (IC_50_). The IC_50_ values, reported as the mean ± SD in Table 2, indicate the concentration of the tested compound that inhibits the growth/viability of the cancer cell population by 50%. In this specific case of an MTT assay, which measures the activity of mitochondrial dehydrogenases (as an indicator of cell viability), the IC_50_ values stand for the concentration that inhibits the metabolic activity of cells by 50%.

In accordance with the docking results, the compounds with *ortho* substitution on the central phenoxy group, namely compounds **2** and **3**, were identified as the most cytotoxic. The greatest potency of **2** and **3** was against HepG2 (IC_50_ = 10.39 and 2.97 μM, respectively), followed by T98G (IC_50_ = 26.82 and 28.34 μM, respectively), and then against HT29 (IC_50_ = 43.91 and 78.32 μM, respectively). A similar trend was observed for the structure-activity relationship (SAR) of **4**, with a *meta* substitution in the central phenoxy group and analogue *para* **5** with a distal dichlorophenyl substitution. It is important to note that, although **4** and **5** were ~1.5- to ~7-fold less potent than **3**, the results obtained in the T98G and HepG2 cells showed that these compounds were still significantly more potent than the reference compound etoposide, with the best results against T98G cells obtained by **4** (IC_50_ = 42.37 μM) and against HepG2 cells by **5** (IC_50_ = 13.87 μM).

The results of the antiproliferative assay for compounds **2**–**5** were subsequently confirmed by staining normal CCD 841 CoTr and the most resistant cancer cells in our study (i.e., HT29) using the May–Grünwald–Giemsa (MGG) method. In general, cytotoxic effects in this assay can be indicated by a thinning of the culture, due to cells detaching from the culture surface, and changes in cells’ morphology can be indicated mainly by cell elongation or shrinkage, as well as cells detaching from each other.

**Figure 8:**
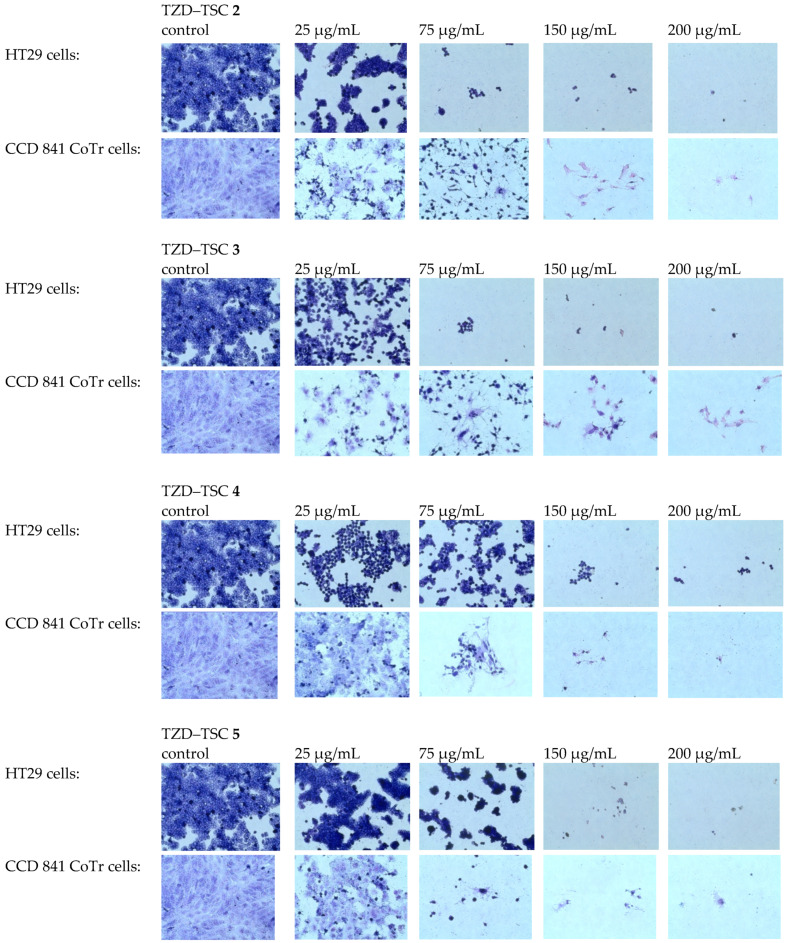
MGG staining. Effects of TZD–TSCs **2**–**5** in a concentration range of 25 to 200 μg/mL on the morphology of tumor (HT29) and normal (CCD 841 CoTr) cells (taken at 40× magnification), in comparison with the control (untreated cells). MGG, May–Grünwald–Giemsa.

We found that the TZD–TSC-induced inhibition of HT29 cell proliferation is associated with a dose-dependent reduction in the number of stained cells (Figure 8), due to the detachment of the cells that are usually considered dead. Thereby, compounds **2**–**5**, at the highest tested concentration of 200 µg/mL, possessed the strongest cytotoxic effect. Due to the exposure of HT29 cancer cells to compounds **2**–**5** in the cultures, effects such as cancer cells’ detachment from each other, their shrinkage, and the loss of intercellular interactions were also detected. Normal cells (CCD 841 CoTr) were also sensitive to the activity of the tested compounds. In accordance with antiproliferative assay results (Table 2), the compounds **4** and **5** were even more cytotoxic towards normal cells than cancerous HT29 cells. At the highest concentration tested (200 µg/mL), the cells shrank, leaving only the cytoplasmic lining connecting neighboring cells. In addition, a reduced number of cells was found, compared to the control.

Since selectivity between tumorigenic and normal cells is the most relevant parameter to detect anticancer potential in vitro [64,65,66], selectivity indices (SI)—calculated by the formula: the IC_50_ value for each compound against CCD 841 CoTr cells divided by the IC_50_ value of each cancer cell line—were then used to quantify this parameter. SI values greater than 1.0 denote the selectivity of the tested compound for cancerous over non-cancerous cells. These results revealed that compound **3**, with a SI of 33.80 in the HepG2 cell line, a SI of 3.54 in the T98G cell line, and a SI of 1.28 in the HT29 cell line, was the most selective. Selectivity indexes for other compounds are presented in Table 2. Although no SAR analysis could be deducted due to limited data thus far, the selectivity of the tested TZD–TSCs for cancerous over non-cancerous cells seems to be tumor-specific; HepG2 cells were most sensitive to the activity of the tested compounds, compared to T98G cells, while HT29 cells were the most resistant. Another observation is that the substitution pattern of the distal benzene ring may have a profound impact on the anticancer potency of TZD–TSCs. While the evidence remains limited, e.g., on the selectivity between tumor cells and normal cells of the same tissue type, further research is imperative to unravel the full anticancer potential of TZD–TSCs.

### 2.5. Cell Cycle Analysis

HDAC inhibitors, in combination therapy with PPARγ agonists, exert synergistic/additive cytotoxic effects on cancer cells, resulting in proliferation arrest and increased cancerous cell apoptosis [24]. Based on this evidence from the literature, in order to explore the molecular mechanisms of the anti-proliferative activity of the investigated group of TZD–TSCs, the effect of the most potent cytotoxic agent, compound **3**, on cell cycle progression was subsequently tested by a cytometry technique.

**Figure 9:**
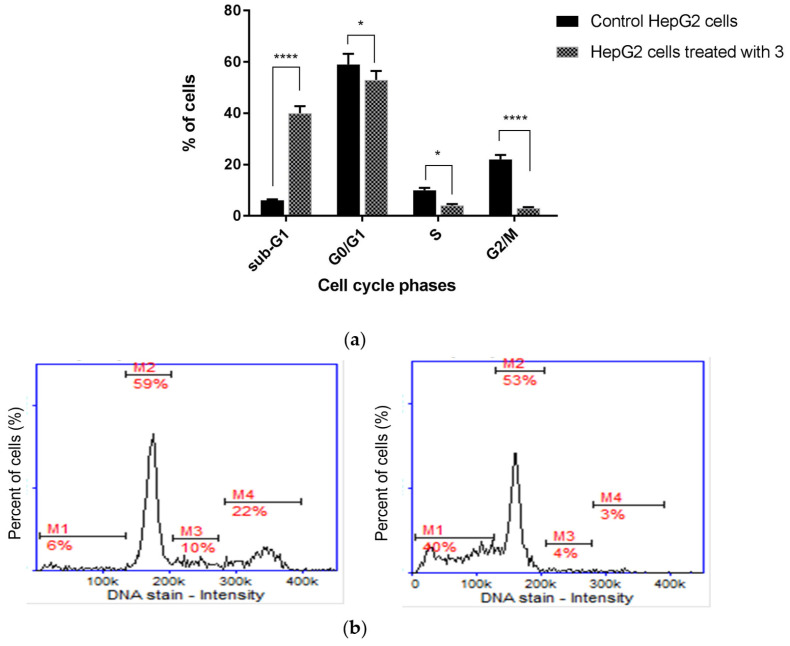
Cell cycle analysis of HepG2 cells (**a**) incubated for 24 h with TZD–TSC 3 at its IC_50_ concentration. Results are expressed as means ± SD. Statistical significance was designated as “****” when *p* < 0.0001 or “*” when *p* < 0.05 (vs. control cells), using ANOVA followed by Tukey’s post hoc test; (**b**) representative histograms of the untreated (control) HepG2 cells (**left**) and HepG2 cells treated with TZD–TSC 3 (**right**). M1, M2, M3, and M4 denote the cells in sub-G1, G0/G1, S, and G2/M phases, respectively.

We found that the inhibition of HepG2 cell growth is associated with cell cycle arrest in the sub-G1 phase (Figure 9). Quantitative analysis of the DNA content revealed significant (*p* < 0.0001) sub-G1 accumulation of HepG2 cells, as their percentage increased from 6.02 ± 0.21% in the control group to 40.36 ± 3.11% after treatment with compound **3**. Diminished DNA content, which is visible as an elevated sub-G1 fraction in cells, is recognized as a marker of apoptotic changes. The identification of apoptosis via sub-G1 phase analysis is based on the principle that LMW DNA fragments (low molecular weight DNA), which are recognized as early signs of cellular apoptosis, are released from cells, which results in an increase in the number of cells with reduced DNA content. This parameter (i.e., DNA content), in turn, can be measured using numerous methods, including cytometry. In summary, cell cycle analysis proved that the superior anticancer activity of TZD–TCS **3** against HepG2 cells was mainly caused by the intense stimulation of apoptosis.

### 2.6. Anti-Metastatic Potential of Thiazolidinedione–Thiosemicarbazones (TZD-TSCs)

Metastasis is a complex process that includes, among others, the migration and invasion of cancer cells through the extracellular matrix (ECM). As a result, cancer cells are able to disseminate to distant organs from primary tumors. It is estimated that metastasis contributes to almost 90% of cancer deaths [67,68]. Therefore, anticancer drugs with anti-metastatic potential constitute a promising approach in current oncology.

**Figure 10:**
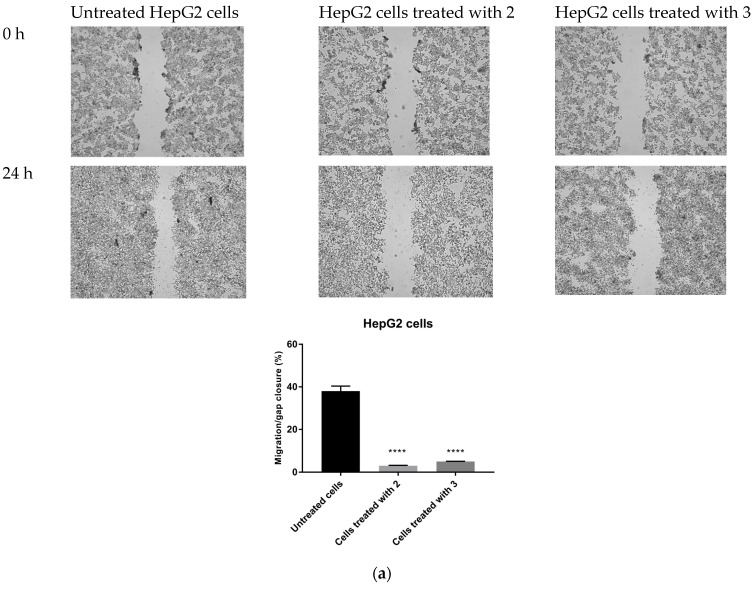

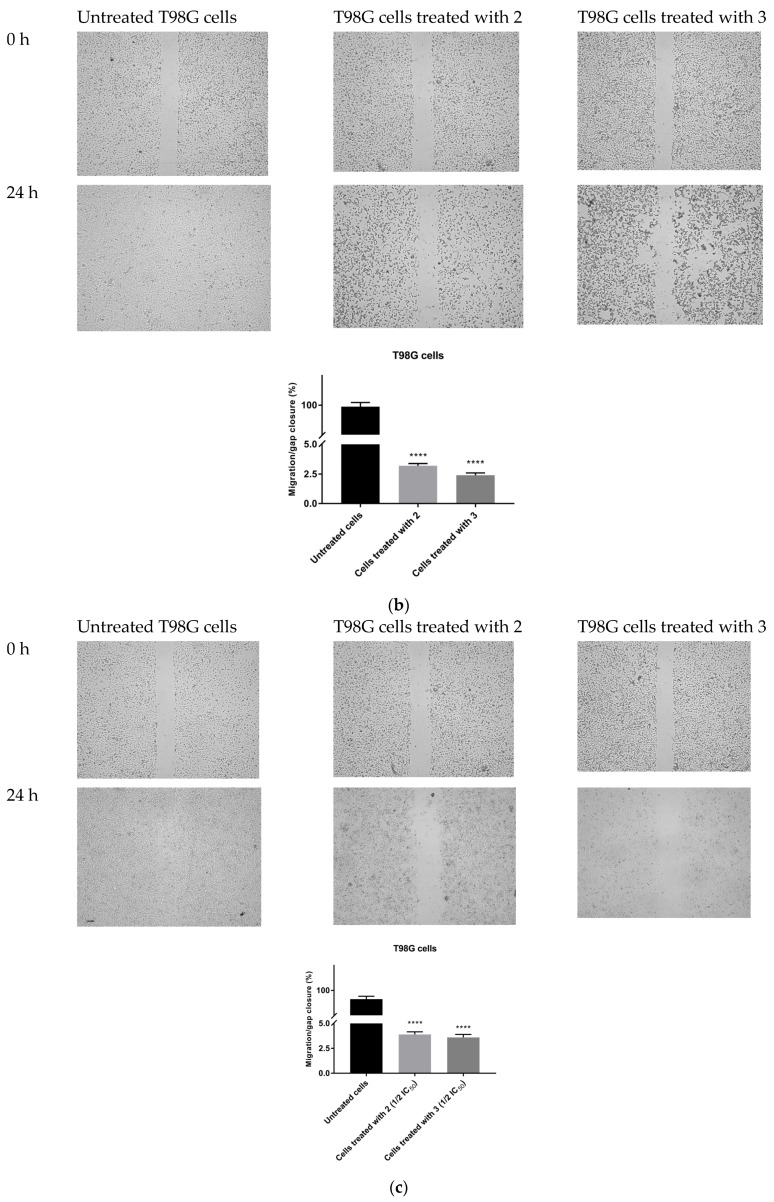
Anti-migratory effect of TZD–TSCs **2** and **3** against HepG2 and T98G cells. Representative images (taken at 20× magnification) and graphs represent: (**a**) HepG2 cells treated with IC_50_ concentrations of **2** (10.39 μM) and **3** (2.97 μM); (**b**) T98G cells treated with IC_50_ concentrations of **2** (26.82 μM) and **3** (28.34 μM); (**c**) T98G cells treated with ½ × IC_50_ concentrations of **2** (13.41 μM) and **3** (14.17 μM). Results were statistically analyzed by ANOVA with a post hoc Dunnett’s test. Statistical significance was designated as **** when *p* < 0.0001 (vs. untreated cells).

**Figure 11:**
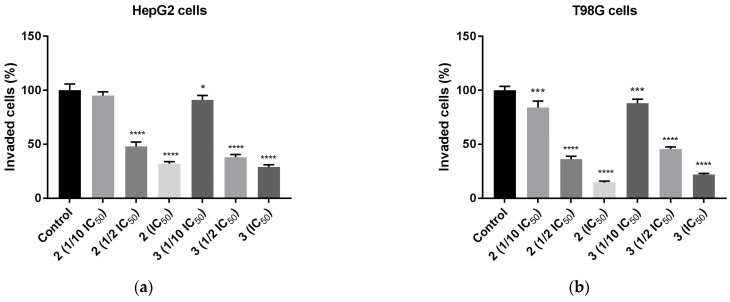
Anti-invasion effect of TZD–TSCs **2** and **3** against HepG2 and T98G cells, measured by ECM cell invasion assay (colorimetric); (**a**) HepG2 cells treated with 1/10 IC_50_, 1/2 IC_50,_ IC_50_ concentrations of **2** and **3**; (**b**) T98G cells treated with 1/10 IC_50_, 1/2 IC_50,_ IC_50_ concentrations of **2** and **3**. Results were statistically analyzed by ANOVA with post hoc Dunnett’s test. Statistical significance was designated as **** when *p* < 0.0001, *** when *p* < 0.001, and * when *p* < 0.05 (vs. control cells). For the IC_50_ values for **2** and **3** against HepG2 and T98G cells, see Table 2.

The possible anti-migratory effect of **2** and **3** was examined against HepG2 and T98G cells, which most effectively responded to TZD–TSC treatment. The ability of cells to cover the scratched area was monitored after 24 h of incubation with compounds **2** and **3**. As shown in Figure 10, the migration of the investigated cancer cells was almost completely suppressed by the treatment with TZD–TSCs at their respective IC_50_ doses against HepG2 and T98G cancer cells (Table 2). Importantly, although T98G cells nearly completely covered the area between the scratched edges after 24 h of incubation, their exposure to even ½ IC_50_ concentrations of **2** (13.41 μM) and **3** (14.17 μM) also resulted in a reduction in the migratory potential of the cancer cells by >90% (Figure 10c). As shown in Figure 11, both **2** and **3** exhibited inhibitory effects against the invasion of HepG2 and T98G cells through an artificial model of the ECM. This anti-invasion effect was observed at a wide range of concentrations. The invasion of T98G cells was statistically significantly inhibited, even after their exposure to 1/10 of the IC_50_ doses of **2** and **3**. In the case of HepG2 cells, only compound **2**, at its lowest concentration tested (i.e., 1/10 of the IC_50_), did not reduce the number of invaded cells. Thus, we can conclude that the anticancer potential of the investigated compounds is enriched by their anti-metastatic effect.

### 2.7. Hemolytic Activity of Thiazolidinedione–Thiosemicarbazones (TZD-TSCs)

Generally, most anticancer agents are delivered parenterally, either by intravenous (IV) infusion or injection. This route of administration is the most direct and ensures 100% bioavailability. However, the ability of the drug molecules to induce hemolysis of the red blood cells (RBCs) is, at the same time, one of the crucial contraindications for IV administration. Therefore, it seems reasonable to test the possible risk of RBC hemolysis at the earliest possible stage of drug development.

**Figure 12:**
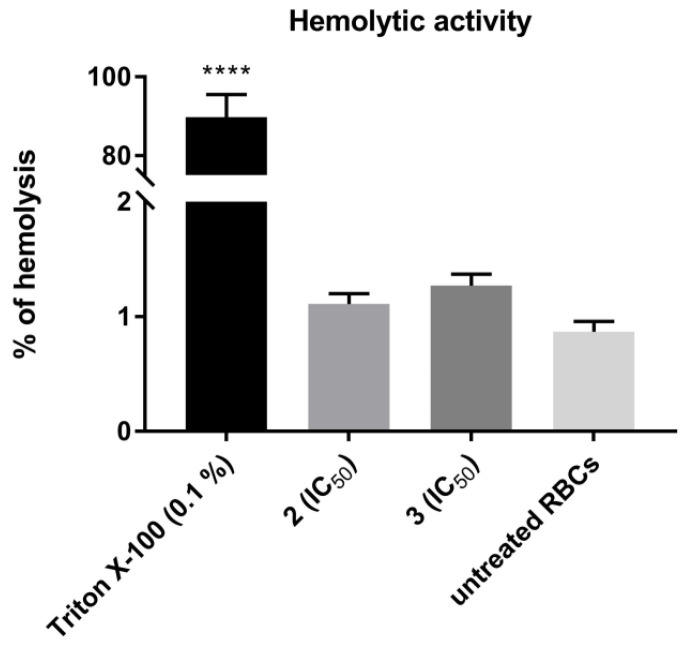
Hemolytic activity of TZD–TSCs **2** and **3** in human red blood cells (RBCs). Each bar expresses the mean ± SD (n = 4). Results were designated as statistically significant (ANOVA with post hoc Dunnett’s test) when *p* < 0.05 (vs. untreated RBCs). Statistical significance was designated as **** when *p* < 0.0001. TZD–TSCs **2** and **3** were tested at the concentrations equal to their IC_50_ values examined for the most sensitive cancer cell line (i.e., HepG2). Triton X-100 was used as a positive control.

The hematologic toxicity of the most cytotoxic TZD–TSCs, compounds **2** and **3,** was tested via in vitro hemolysis assay, following a 30 min treatment of human RBCs at 37 °C with these compounds. As shown in Figure 12, no statistically significant increases in the levels of free hemoglobin released to the medium were observed in either case, thereby indicating that **2** and **3**, at the concentrations equal to their IC_50_ values examined for the most sensitive cancer cell line (i.e., HepG2), showed no specific hemolytic activity.

### 2.8. Influence of Thiazolidinedione–Thiosemicarbazones (TZD-TSCs) on Nitric Oxide Production

**Figure 13:**
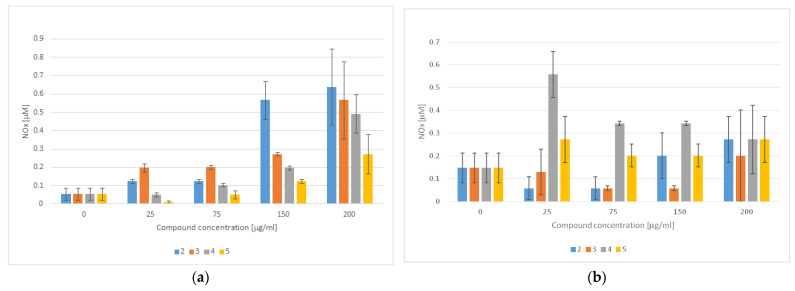
NO detection using the Griess method in culture supernatants obtained after the incubation of (**a**) CCD 841 CoTr cells and (**b**) HT29 cells with TZD–TSCs **2**–**5** for 3 h (mean ± SD of three independent experiments). Nitrite concentration was determined by comparing the test samples’ absorbance values to a standard curve generated with 0.5–25 μM of NaNO_2_.

In addition to the role of PPARγ in adipogenesis, strong evidence supports the theory that PPARγ activation also regulates endothelial cell homeostasis. Consistently, the beneficial effects of TZD-based compounds on endothelial cell functions and activation have been well-recognized in the literature. For instance, in addition to suppressing the effects of TZD-based compounds on the gene expression of adhesion molecules, it has been proven that TZDs enhance nitric oxide (NO) production in endothelial cells and inhibit reactive oxygen species (ROS) production [69]. Apart from the multiple physiological roles of NO, ranging from the regulation of the vascular tone to neurotransmission, it has also been proven that NO has antibacterial properties and plays an important role in carcinogenesis [70]. In fact, unlike nanomolar and even lower concentrations of NO, which are known to accelerate tumor progression and invasiveness, micromolar concentrations of NO promote cytotoxicity and inhibit P-glycoprotein activity, thus representing a promising option for anticancer therapy [71,72]. We therefore evaluated the effect of TZD–TSCs **2**–**5** on NO production, both in cancerous (HT29) and noncancerous (CCD 841 CoTr) cells. As shown in Figure 13a, the amounts of NO produced in the normal colon epithelial cells increased in a manner dependent on the compounds’ concentration. Consequently, treatment with the highest concentration of TZD–TSC **2**–**5** (200 μg/mL) was the most effective at increasing the total NO concentration. Compound **4**, with *meta* phenoxy substitution, was the most effective ([NO_2_^−^] = 0.639 μM), followed by *ortho* phenoxy analogues **2** ([NO_2_^−^] = 0.566 μM) and then **3** ([NO_2_^−^] = 0.492 μM), whereas compound **5**, with *para* phenoxy substitution, was the least effective ([NO_2_^−^] = 0.272 μM). In respect to the cancerous HT29 cell line, in turn, the tumor cells were seen to produce the highest amounts of NO (0.558 μM) after incubation with **4**, at the lowest tested concentration of 25 μg/mL. Higher concentrations of **4** resulted in decreased NO production, probably due to the decreased viability of the cells. For the remaining compounds, however, a lack of correlation between NO production and the inhibition of cancer cell proliferation was seen. Indeed, as shown in Figure 13b, for the most cytotoxic *ortho* isomers **2** and **3**, the highest amounts of NO (0.272 and 0.201 μM, respectively) were detected after the incubation of HT29 cells with these compounds at the highest tested concentration of 200 μg/mL, while the incubation of cancer cells with **5** resulted in comparable amounts of NO (~0.272 μM), at both the lowest and the highest concentrations of the compound tested.

Due to the lack of correlation between NO production and TZD–TSC (**2**–**5**)-induced toxicity in noncancerous CCD 841 CoTr cells and cancerous HT29 cells (for IC_50_ values see Table 2), we presume that the observed cytotoxic effect was NO-independent and, thus, not accompanied by nitrosative stress [70].

### 2.9. Antioxodant Properties of Thiazolidinedione–Thiosemicarbazones (TZD-TSCs)

TZD-based compounds have a dual role in reactive oxygen species (ROS) generation; on one hand, they can promote ROS production, inducing oxidative stress in vitro in different cancer cell types [55]. Oxidative stress, acting similarly in a manner to nitrosative stress, can then affect homeostasis and alter protein function, inducing a cytotoxic effect that involves the activation of apoptotic pathways or the inhibition of resistance to anticancer treatments [73,74,75]. On the other hand, TZD-based compounds proved their antioxidant properties, confirming their role as antiradical agents [55,76]. Therefore, in the subsequent stage of our study, the antioxidant properties of TZD–TSCs **2**–**5** were evaluated.

Antioxidant activity is a complex procedure, usually happening through several multiple or predominant chemical mechanisms (i.e., hydrogen atom transfer (HAT), single electron transfer (SET), and the ability to chelate transition metals), and it is influenced by many factors, which cannot be fully described with one single method. Therefore, it is important to perform more than one type of measurement to take into account the various mechanisms of antioxidant action. In this study, to assess the antioxidant activity of TZD–TSCs **2**–**5**, two complementary tests were used: a DPPH (2,2-diphenyl-1-picrylhydrazyl) free radical-scavenging ability assay, that detects the HAT and SET mechanisms, and a FRAP (ferric reducing antioxidant power) assay, that prefers the SAT mechanism [77]. The antioxidant powers of **2**–**5** were compared to standard antioxidants, such as the water-soluble synthetic analogue of vitamin E (Trolox) and ascorbic acid (vitamin C).

**Figure 14:**
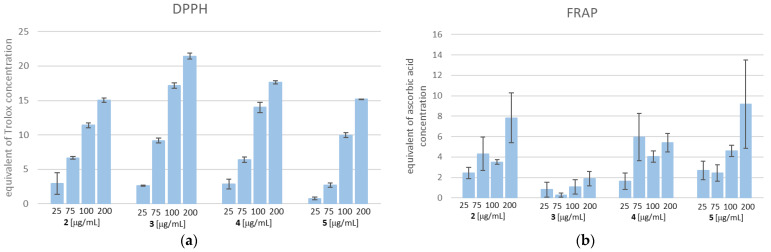
(**a**) DPPH free radical scavenging activity. The results for TZD–TSCs **2**–**5** are presented as reduction values (μg/mL), corresponding to the appropriate concentration of Trolox; (**b**) ferric-reducing antioxidant power (FRAP) assay. The results for TZD–TSCs **2**–**5** are presented as reduction values (μg/mL) corresponding to the appropriate concentration of ascorbic acid. DPPH, 1,1-diphenyl-2-picrylhydrazyl. For details, see Table S1.

As shown in Figure 14, TZD–TSCs **2**–**5** possess both a free radical scavenging ability and the ability to reduce iron ions. With respect to the DPPH method, the scavenging activities of **2**–**5** were dose-dependent (14A). Compound **3**, at the highest tested concentration of 200 µg/mL, possessed the strongest free-radical scavenging effect, equal to 21.439 µg/mL of Trolox. Also, for the remaining compounds, the highest DPPH radical reducing activity was observed when the highest tested concentration of 200 µg/mL was used. Among them, the highest DPPH reducing capacity was recorded for **4** (equal to 17.638 µg/mL of Trolox), followed by **5** (equal to 15.177 µg/mL of Trolox), and then **2** (equal to 15.068 µg/mL of Trolox). It is important to note that the SAR trend observed in the DPPH assay does not reflect the results obtained from FRAP. Indeed, as shown in Figure 14b, the most effective compound in the DPPH assay, compound **3**, at the concentration of 200 μg/mL, showed the weakest reducing activity of the Fe^3+^ ion, equal to 1.89 µg/mL of ascorbic acid, whereas the same concentration of **5** exerted the strongest ferric-reducing power (FRAP), equal to ascorbic acid at 9.19 µg/mL. Moreover, unlike the DPPH method, the lack of dose-dependent ferric-reducing potency was observed for all tested compounds.

Although the SAR trend in antioxidant capacities estimated from the DPPH assay correlated poorly with those assayed by the FRAP method, one can sum up that TZD–TSCs **2**–**5** are characterized by antioxidant properties and, as a result, have the potential to decrease, rather than increase, oxidative stress. Therefore, we presume that their cytotoxic effect is rather ROS-independent, thus not accompanied by oxidative stress.

## 3. Materials and Methods

### 3.1. Molecular Docking Studies

Docking was performed using the FlexX, as implemented in the LeadIT software package (LeadIT version 2.3.2; BioSolveIT GmbH, Sankt Augustin, Germany, 2017). Four crystal structures, PDB IDs: 2VQJ, 4CBY, 3SFF, and 6DGR, were obtained from RCSB Protein Data Bank. All steps of ligand and receptor preparation were carried out using the default settings in BioSolveIT’s LeadIT software. The binding sites were defined to include residues within a 6.5 Å radius around the native ligand. Soft docking (allowing for a volume overlap up to 100 Å^3^) was performed. The clash factor was set to 0.1. Other parameters were set to the default. The conformation with most favorable binding score was next selected for detailed evaluation of binding site interactions. For 2D visualization, the PoseView dock widget, as implemented in LeadIT software, version 2.3.2, was used. According to the PoseView dock widget, the green curves represent the hydrophobic contacts between at least three different ligand atoms and their surrounding residues.

### 3.2. Chemistry

The TZD–TSC hybrid molecules **2**–**5** were resynthesized using previously reported procedures [44]. All commercial reactants and solvents were purchased from either Alfa Aesar (Kandel, Germany) or Tokyo Chemical Industry Co. (Tokyo, Japan) at the highest purity and used without further purification. Melting points and NMR spectra were found to be in accordance with data from the literature. NMR spectra are presented in Supplementary Materials as Figures S1–S8.

### 3.3. General Procedure for the Synthesis of Thiazolidinedione–Thiosemicarbazone (**2–5**) Hybrid Molecules

To a mixture of 0.001 mol of corresponding formylphenyl (2,4-dioxothiazolidin- 5-ylidene)acetate and 0.001 mol of corresponding 4-(chlorophenyl)thiosemicarbazide, 5 mL of ethanol was added. The reaction mixture was heated under reflux for 15 min. After cooling, the precipitate was filtered off and washed with ethanol. After drying, the precipitate was recrystallized from appropriate solvents, i.e., butanol for compounds **2**, **3**, and **5** and DMF:H_2_O (2:1) for compound **4**.

### 3.4. Cell Cultures

Human colon adenocarcinoma cells, HT29 (ATCC no. HTB-38), derived from a grade I tumor, were cultured in roswell park memorial institute Medium (RPMI) 1640 medium, supplemented with 10% fetal calf serum (FCS) (GibcoTM, Paisley, UK) and antibiotics (100 U/mL penicillin and 100 μg/mL streptomycin) (Sigma, St. Louis, MO), at 37 °C in a humidified atmosphere with 5% CO_2_. Human normal colon epithelial cells, CCD 841 CoTr (ATCC no. CRL-1807), were cultured in RPMI 1640 + dulbecco’s modified eagle medium (DMEM) (1:1) medium (Sigma, St. Louis, MO), supplemented with 10% FCS and antibiotics, at 34 °C in a 5% CO_2_/95% air atmosphere [78]. Human glioblastoma T98G cells were cultured in Dulbecco’s Modified Eagle’s Medium (DMEM) (Sigma Aldrich, St. Louis, MO, USA), supplemented with 10% fetal bovine serum (FBS), penicillin (100 U/mL), and streptomycin (100 μg/mL) [79]. Human hepatocellular carcinoma cells, HepG2, were maintained in Eagle’s Minimum Essential Medium, supplemented with 10% FBS, penicillin (100 U/mL), and streptomycin (100 μg/mL) [44].

### 3.5. MTT Assay

After 24 h of incubation of the cells with the tested compounds in 100 μL of culture medium, MTT solution (5 mg/mL, 25 μL/well) was added and incubated for an additional 3 h. The purple crystals of formazan which formed in the medium were solubilized overnight in 10% sodium dodecyl sulphate (SDS) in a 0.01 M HCl mixture. The product was quantified spectrophotometrically by measuring its absorbance at 570 nm using an Emax Microplate Reader (Molecular Devices Corporation, Menlo Park, CA, USA) [78]. A primary single dose screening of the tested compounds was performed at a concentration of 200 μg/mL, and those that elicited a greater than 80% inhibition of cell proliferation were analyzed at several concentrations to find their half-maximal inhibitory concentration (IC_50_).

### 3.6. May–Grünwald–Giemsa (MGG) Staining

The cells were incubated in 24-well plates in 1 mL of culture medium, supplemented with the tested compounds at the concentrations of 25, 75, 150, and 200 μg/mL, respectively. After 24 h of incubation (at 37 °C in a humidified 5% CO_2_/95% air), the medium was discarded and the cell cultures were rinsed with RPMI 1640 medium and stained with May–Grünwald (MG) stain for 5 min, followed by staining for another 5 min with MG diluted in an equal quantity of water. The MG was removed and the Giemsa reagent (diluted 1:20 in water) was added to the cells, which were next incubated at room temperature for 15 min. Thereafter, the cells were rinsed three times with water, dried, and subjected to microscopic observations (Olympus, BX51; Olympus, Tokyo, Japan) [78].

### 3.7. Cell Cycle Analysis

A cell cycle analysis of HepG2 cells, pretreated with compound **3** at a concentration of IC_50_ (2.97 ± 0.39 μM), was carried out using a NucleoCounter NC-3000 Image Cytometer (ChemoMetec, Lillerod, Denmark). HepG2 cells were seeded on 6-well culture plates (Corning Inc., NY, USA) at the density of 1 × 10^5^ cells/mL and cultured in the respective medium at 37 °C in a humidified atmosphere of 5% CO_2_. When the cells reached approximately 80–90% confluence, they were treated with **3**, at a concentration of IC_50_, for 24 h. Subsequently, the cells were detached with trypsine, suspended in 250 μL of lysis buffer (Solution 10), supplemented with DAPI (10 μg/mL), and incubated for 5 min at 37 °C. Next, stabilization buffer (Solution 11) was added and the obtained cell suspension was applied on a NC slide and analyzed using a NucleoCounter NC-3000 Image Cytometer equipped with NucleoView software (https://chemometec.com/software/nucleoview/ (accessed on 20 November 2023). Experiments were repeated three times and the measurements in each experiment were run in duplicate [80].

### 3.8. Cell Migration Assay

Cell migration ability was evaluated according to a wound healing assay. On the day of the experiment, HepG2 and T98G cells were collected from monolayers using trypsin/EDTA and seeded on 6-well plates at a density of 1 × 10^5^ cells mL^−1^. After 24 h, when the confluence of cells reached about 80%, a vertical linear scratch was created in the monolayer with a sterile pipette tip. The cells were washed three times with a phosphate-buffered saline (PBS) to remove all cellular debris. Subsequently, the cells were cultured in a serum-free medium, containing compounds **2** or **3** in the concentrations equal to their IC_50_ or ½ IC_50_ values, as examined for HepG2 and T98G cancer cell lines (data provided in Table 2).Images of the scratch were taken after 0 h and 24 h using an Olympus CKX53 microscope coupled with a XM10 digital camera (Olympus). The experiments were performed at least in duplicate. The scratch area at the beginning of the experiment (0 h) was considered to be 100%. The open wound area was measured with the NIH ImageJ software version 1.53t (Bethesda, Rockville, MD, USA). The percentage of wound closure was calculated using the following formula [81]:

$$Wound\;closure\;\left(\%\right)=\frac{open\;wound\;area\;at\;0\;h\;-open\;wound\;area\;at\;24\;h\;or\;48\;h}{open\;wound\;area\;at\;0\;h}\times 100\%\;$$

### 3.9. Anti-Invasion Assay

The anti-invasion effects of **2** and **3** were examined using a Cell Invasion Assay Kit (ECM550, CHEMICON—Millipore Co., Billerica, MA, USA) [82]. In brief, 10^5^ tumor cells were suspended in medium with 10% FBS and plated into the upper chamber, containing 8 μm pore size polycarbonate membrane precoated with ECMatrix, followed by filling the lower chamber with the same medium, enriched with the investigated compounds. Afterwards, cells were incubated for 24 h and allowed to invade towards the bottom chamber (with medium + 10% FBS). The non-invading cells on the upper membrane surface were removed with a cotton swab and the invasive cells, i.e., cells attached to the lower membrane surface, were stained with 0.1% crystal violet. For colorimetric quantitation, the stained cells were dissolved in 10% acetic acid (200 μL/well) and the obtained solution was transferred to a 96-well plate for spectrometric measurements, at a wavelength of 560 nm.

### 3.10. Hemolytic Activity Evaluation

Human red blood cell (RBC) concentrate was obtained from the Regional Blood Donation and Transfusion Centre (Lublin, Poland). The RBC concentrate (5 mL) was washed three times with sterile PBS and centrifuged at 500 g for 3 minutes. The obtained pellet was suspended in sterile PBS to obtain a 2% suspension, which was subsequently mixed with 1 mL of solution of TZD–TSCs **2** and **3**. TZD–TSCs **2** and **3** were tested at the concentrations equal to their IC_50_ values (43.91 ± 1.74 and 2.97 ± 0.39μM, respectively), as examined for the most sensitive cancer cell line, i.e., HepG2. The mixtures were incubated at 37 °C for 30 min and, subsequently, centrifuged at 1400× *g* for 10 min. The amount of free hemoglobin in the supernatants was measured spectrophotometrically at 405 nm. Negative and positive controls were performed by incubating RBCs with sterile PBS and 0.1% Triton-X, respectively. Each experiment was run in triplicate [45].

### 3.11. Nitric Oxide (NO) Measurements

Nitrate, i.e., a stable end product of NO, was measured in culture supernatants with a spectrophotometric method based on the Griess reaction. Culture supernatants were collected from the cell cultures treated with specific concentrations of the tested compounds (25, 75, 150, and 200 μg/mL, respectively). Briefly, 100 μL of the culture supernatant was placed in 96-well flat-bottomed plates in triplicate and incubated with 100 μL of Griess reagent (1% sulfanilamide/0.1% N-(1-naphthyl)ethylenediamine dihydrochloride) in 3% H_3_PO_4_ at room temperature for 10 min. The optical density was measured at 550 nm using a microplate reader. A standard curve was prepared using 0.5–25 μM sodium nitrite (NaNO_2_) for calibration [78].

### 3.12. DPPH Free Radical Scavenging Test

The free radical scavenging activity of terpene was measured by the DPPH assay [83]. Briefly, 100 μl of DPPH solution (0.2 mg/mL in ethanol) was added to 100 μL of the tested compound concentrations (25, 75, 150, and 200 μg/mL, respectively). Trolox, at increasing concentrations (1–50 μg/mL), was used as a standard. After 20 min of incubation at room temperature, the absorbances of the solutions were measured at 515 nm. The lower the absorbance, the higher the free radical scavenging activity of the compound. The activity of each compound was determined by comparing its absorbance with that of the standard.

The ability of the compounds to scavenge the DPPH radical was calculated by the following formula:

DPPH scavenging effect (%) = [(X_control_ − X_compound_/X_control_) × 100],

where X_control_ is the absorbance of the control and X_compound_ is the absorbance in the presence of TZD-TSCs preparations.

### 3.13. Ferric-Reducing Antioxidant Power Assay

Each compound concentration (25, 75, 150, and 200 μg/mL, respectively) was dissolved in Milli-Q water and mixed with an equal volume of 0.2 M sodium phosphate buffer (pH 6.6) and 1% potassium ferricyanide. The mixture was incubated for 30 min at 37 °C. Thereafter, 10% trichloroacetic acid (*w*/*v*) was added and the mixture was centrifuged at 1000 g for 5 min. One mL of the upper layer was mixed with an equal volume of Milli-Q water and 0.1% ferric chloride. The absorbance was read at 700 nm using an EL800 Universal Microplate Reader (BioTek Instruments, Winooski, VT, USA). Ascorbic acid (0–150 μg/mL) was used as a positive control [78].

### 3.14. Statistical Analysis

The results are presented as means ± SD from three experiments. Data were analyzed using a one-way ANOVA with a Dunnett’s post hoc test. Differences of *p* ≤ 0.05 were considered significant.

## 4. Conclusions

This report outlines our initial assessment of the anticancer effectiveness of molecular hybrids featuring the thiazolidinedione–thiosemicarbazone (TZD-TSC) scaffold. The evaluation of their antiproliferative activity across three cancer cell lines and one noncancerous cell line revealed that TZD–TSCs **2**–**5** exhibited varying degrees of cytotoxicity, ranging from moderate to high. In several instances, their potency surpassed that of the clinical drug etoposide. Among these compounds, **3**—previously identified as a potent antibacterial agent—demonstrated the most promising anticancer potential. It displayed the highest selectivity indices against all tested cancer cell lines, while exhibiting no specific hemolytic activity. Analysis of the cell cycle indicated that the superior anticancer activity of compound **3** primarily stemmed from its strong induction of apoptosis. Additionally, treatment with compound **3** induced notable alterations in the morphology of cancer cells and further inhibited their migratory capabilities. Docking studies corroborated that TZD–TSCs **2**–**4** effectively bound to both HDAC4/8 and PPARγ LBD, demonstrating comparable or even superior calculated free binding energies in comparison to the native ligands.

While the evidence remains somewhat limited, further research is imperative to unravel the full anticancer potential of compound **3** and to validate the experimentally proposed mode of action. Nevertheless, the preliminary findings from this combined in silico and in vitro study underscore the promising role of TZD–TSCs in the rational design of dual-functioning antibacterial and anticancer chemotherapeutics. 

## Figures and Tables

**Table 1:** Docking scores of TZD–TSCs **2**–**5** for HDAC4, HDAC8, and PPARγ LBD.

TZD–TSC		HDAC4_o_^1^	HDAC4_c_^2^	HDAC8	PPARγ LBD
**2**	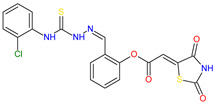	−25.4	−18.5	−21.9	−25.8
**3**	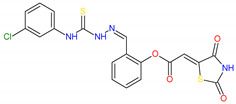	−27.0	−18.5	−20.1	−25.5
**4**	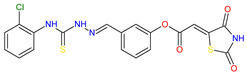	−27.8	−18.0	−21.1	−28.5
**5**	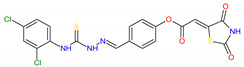	– ^3^	–	–	−27.2
Native Ligands:		−27.2	−28.1	−22.3	−16.1

^1^ HDAC4_o_—open conformation. ^2^ HDAC4_c_—closed conformation. ^3^—a lack of interactions.

**Table 2:** Anti-proliferative effects of TZD–TSCs **2**–**5.**

TZD-TSC	Cell Line	IC_50_ [μM]	SI ^1^
**2**	T98G	26.82 ± 1.54	1.66
	HepG2	10.39 ± 0.72	4.30
	HT29	43.91 ± 1.74	1.02
	CCD 841 CoTr	44.65 ± 2.91	
**3**	T98G	28.34 ± 2.21	3.54
	HepG2	2.97 ± 0.39	33.80
	HT29	78.32 ± 5.47	1.28
	CCD 841 CoTr	100.39 ± 4.82	
**4**	T98G	42.37 ± 2.23	2.06
	HepG2	20.13 ± 1.19	4.34
	HT29	116.99 ± 9.37	0.75
	CCD 841 CoTr	87.31 ± 5.06	
**5**	T98G	93.63 ± 3.45	0.79
	HepG2	13.87 ± 0.59	5.35
	HT29	118.78 ± 10.72	0.62
	CCD 841 CoTr	74.17 ± 2.30	
Etoposide	T98G	>160.00	n.d.
	HepG2	61.86 ± 2.97	n.d.
	HT29	160.55 ± 8.51	n.d.
	CCD 841 CoTr	n.d. ^2^	

^1^ SI—selectivity index. ^2^ n.d.—not determined.

## Data Availability

Data are contained within the article.

## Supplementary Materials

The following supporting information can be downloaded at: https://www.mdpi.com/article/10.3390/ijms242417521/s1.
