# Supplementary material for: Combined In Silico and In Vitro Analyses to Assess the Anticancer Potential of Thiazolidinedione–Thiosemicarbazone Hybrid Molecules
Source: Int J Mol Sci. 2023 Dec 15;24(24):17521. doi: 10.3390/ijms242417521 (PMC10743653; doi:10.3390/ijms242417521)
Supplement: Supplementary file 1 [file ijms-24-17521-s001.zip › ijms-2721331-supplementary.pdf]

Combined *in silico* and *in vitro* analyses to assess the anti-cancer potential of thiazolidinedione-thiosemicarbazone hybrid molecules

Agata Paneth[1], Barbara Kaproń[2], Tomasz Plech[3], Roman Paduch[4], Nazar Trotsko[1*], and Piotr Paneth[5*]

[1] Chair and Department of Organic Chemistry, Faculty of Pharmacy, Medical University of Lublin, Lublin, Poland
[2] Department of Clinical Genetics, *Faculty* of *Medicine,* Medical University of Lublin, Lublin, Poland
[3] Department of Pharmacology, *Faculty* of *Health* Sciences, Medical University of Lublin, Lublin, Poland
[4] Department of Virology and Immunology, Institute of Biological Sciences, Faculty of Biology and Biotechnology, Maria Curie-Skłodowska University, Lublin, Poland
[5] Institute of Applied Radiation Chemistry, Faculty of Chemistry, Lodz University of Technology, Poland
* Correspondence: piotr.paneth@p.lodz.pl (P.P.) and nazar.trotsko@umlub.pl (N.T.)

Supplementary Materials

Table S1. DPPH free radical scavenging activity and Ferric-reducing antioxidant power (FRAP) of TZD-TSCs **2-5**..

3

Figure S1. ^{1}H NMR spectra for **2**..

4

Figure S2. ^{13}C NMR spectra for **2**..

5

Figure S3. ^{1}H NMR spectra for **3**..

6

Figure S4. ^{13}C NMR spectra for **3**..

7

Figure S5. ^{1}H NMR spectra for **4**..

8

Figure S6. ^{13}C NMR spectra for **4**..

9

Figure S7. ^{1}H NMR spectra for **5**..

10

Figure S8. ^{13}C NMR spectra for **5**..

11

Table S1. DPPH free radical scavenging activity and Ferric-reducing antioxidant power (FRAP) of TZD-TSCs **2**-**5**.

**Samples**	**Concentration [μg/mL]**			
	**25**	**75**	**150**	**200**
**DPPH assay (reduction values corresponding to the appropriate concentration of Trolox, μg/mL)**				
**2**	2.942±1.587	6.670±0.205	11.412±0.358	15.068±0.307
**3**	2.616±0.102	9.168±0.358	17.167±0.410	21.439±0.410
**4**	2.834±0.717	6.381±0.410	13.982±0.717	17.638±0.256
**5**	0.768±0.191	2.689±0.307	9.964±0.358	15.177±0.051
**FRAP assay (reduction values corresponding to the appropriate concentration of ascorbic acid, μg/mL)**				
TZD-TSC **2**	2.43±0.54	4.32±1.62	3.51±0.24	7.84±2.43
TZD-TSC **3**	0.81±0.72	0.27±0.21	1.08±0.72	1.89±0.72
TZD-TSC **4**	1.62±0.81	5.95±2.30	4.05±0.54	5.41±0.90
TZD-TSC **5**	2.70±0.90	2.43±0.81	4.59±0.54	9.19±4.32

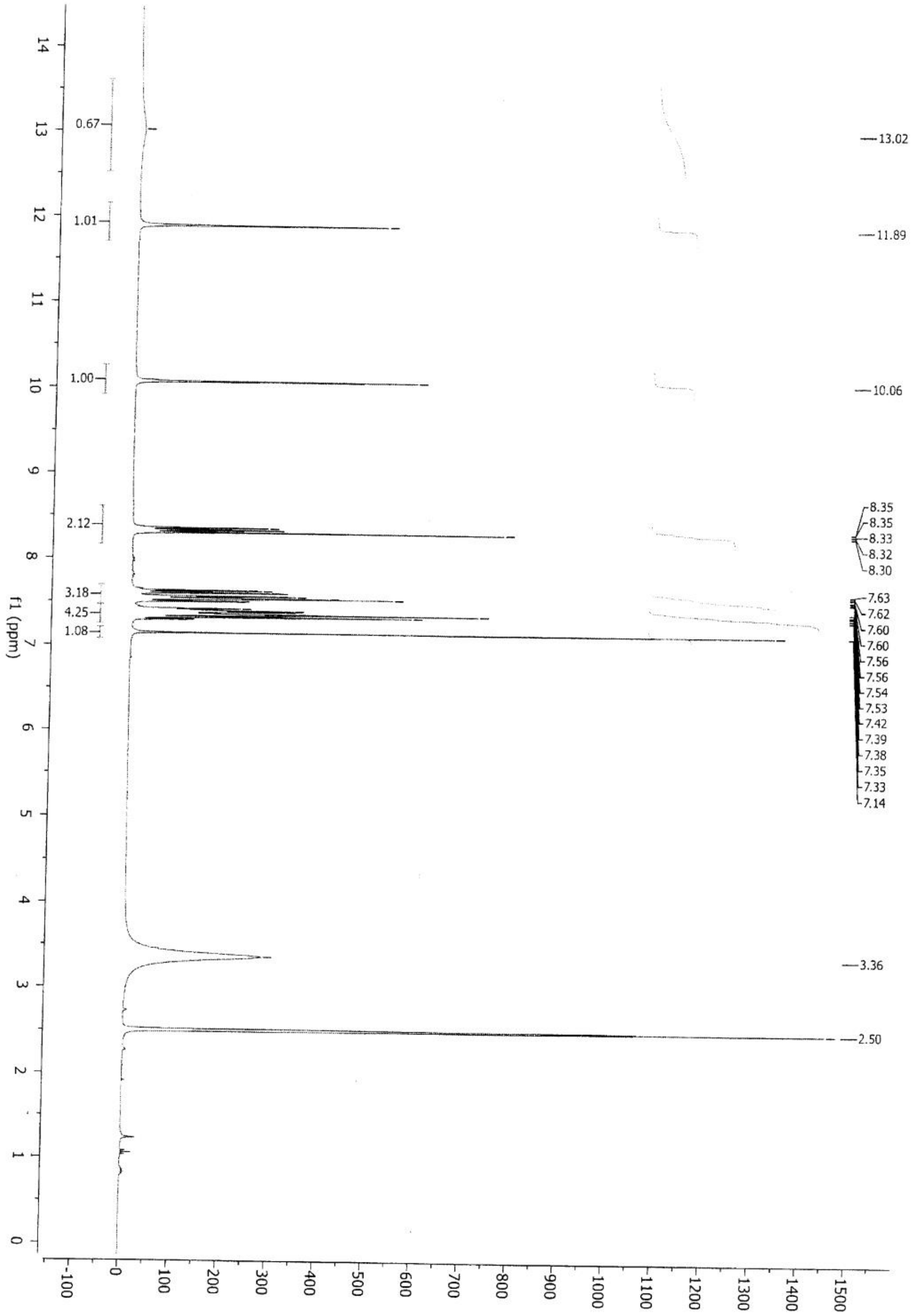

Figure S1. ^{1}H NMR spectra for **2**

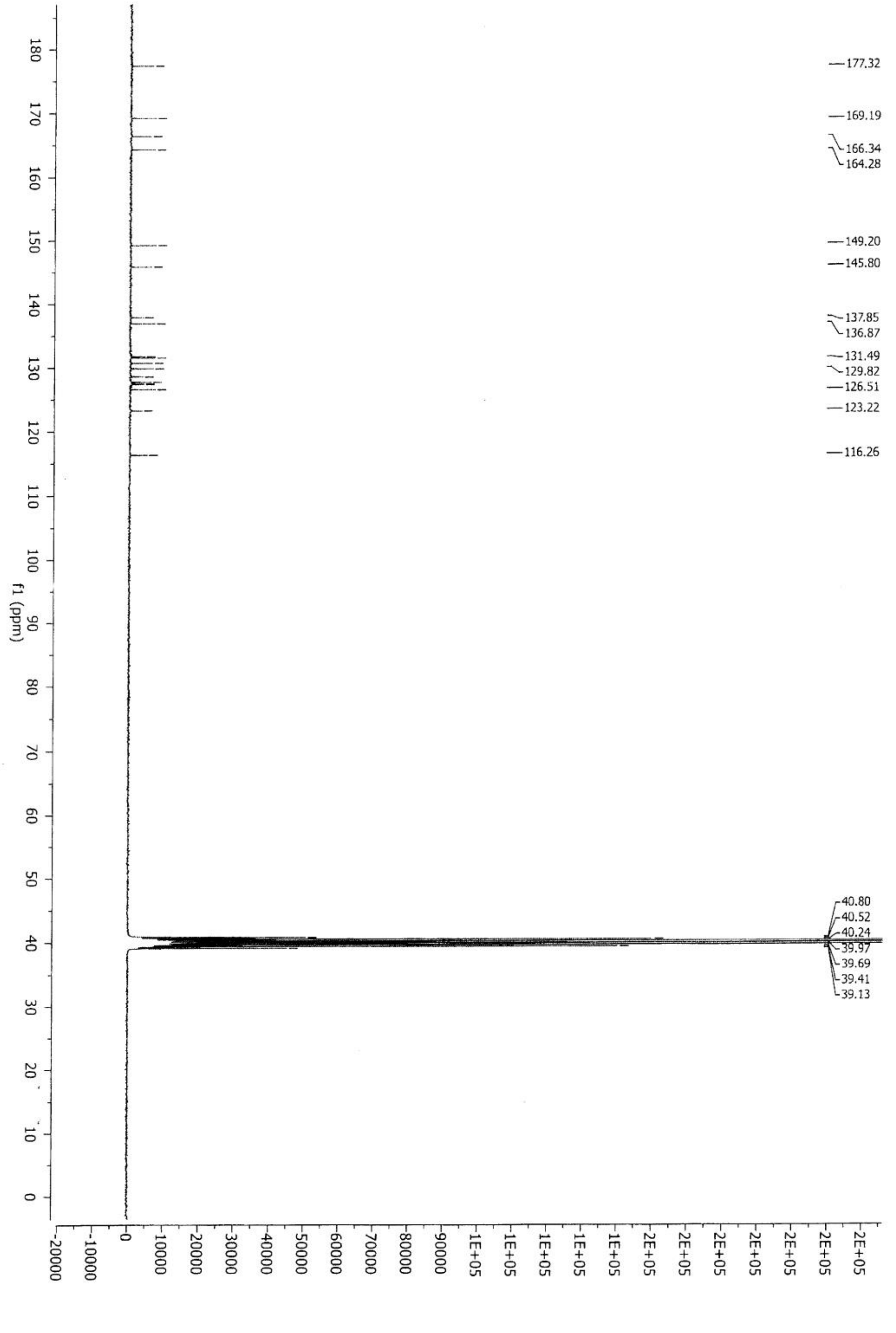

Figure S2. ^{13}C NMR spectra for **2**

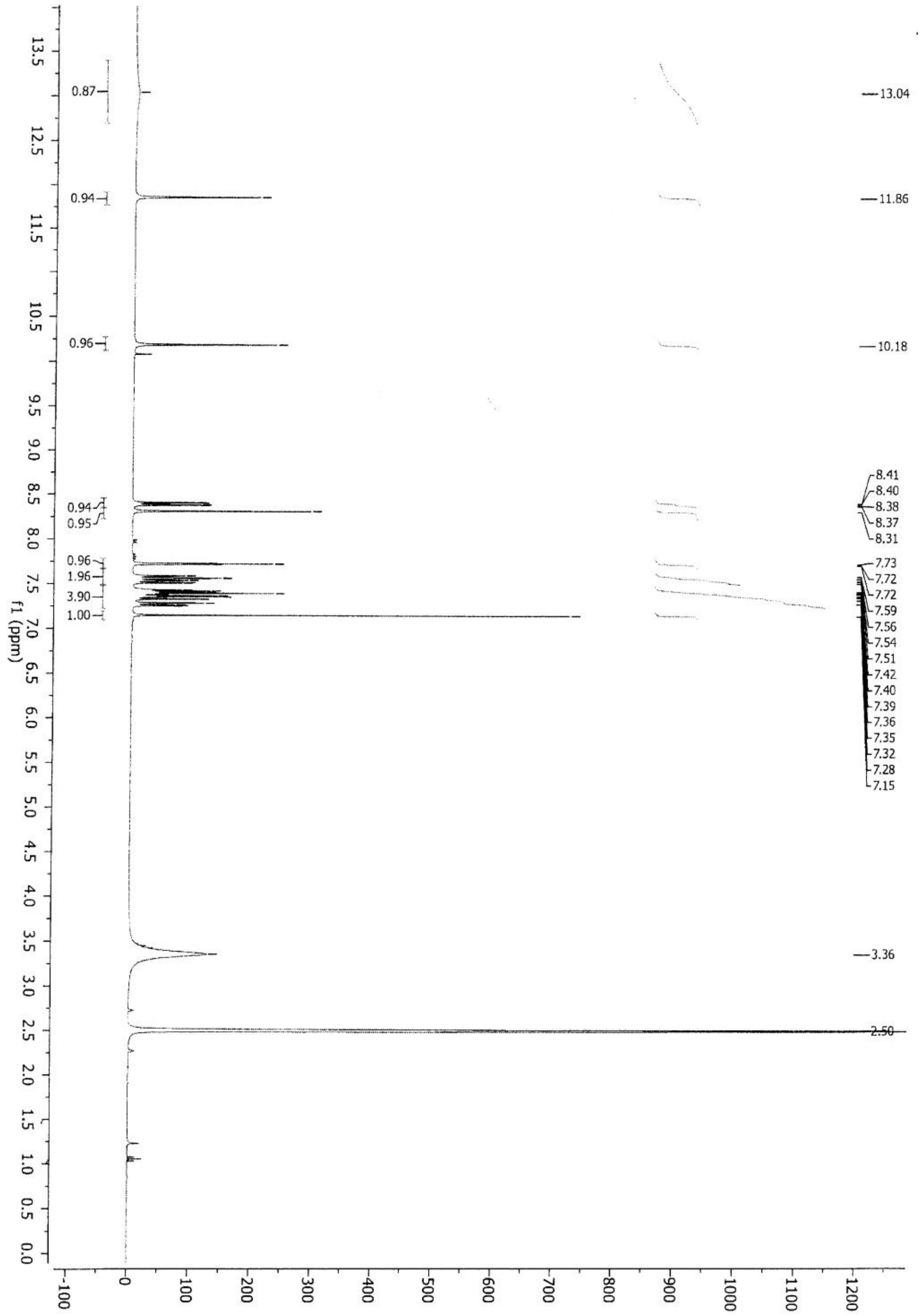

Figure S3. ^{1}H NMR spectra for **3**

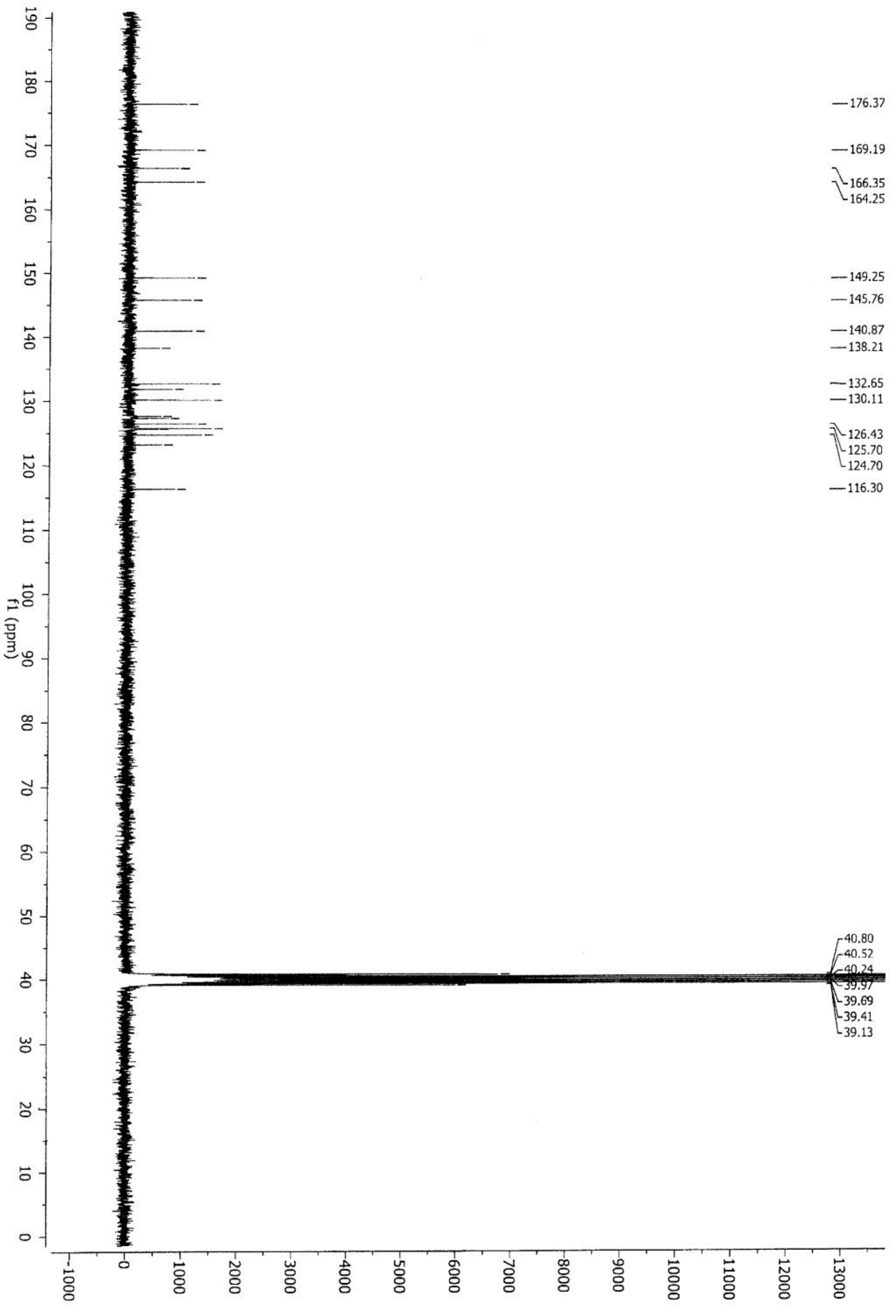

Figure S4. ^{13}C NMR spectra for **3**

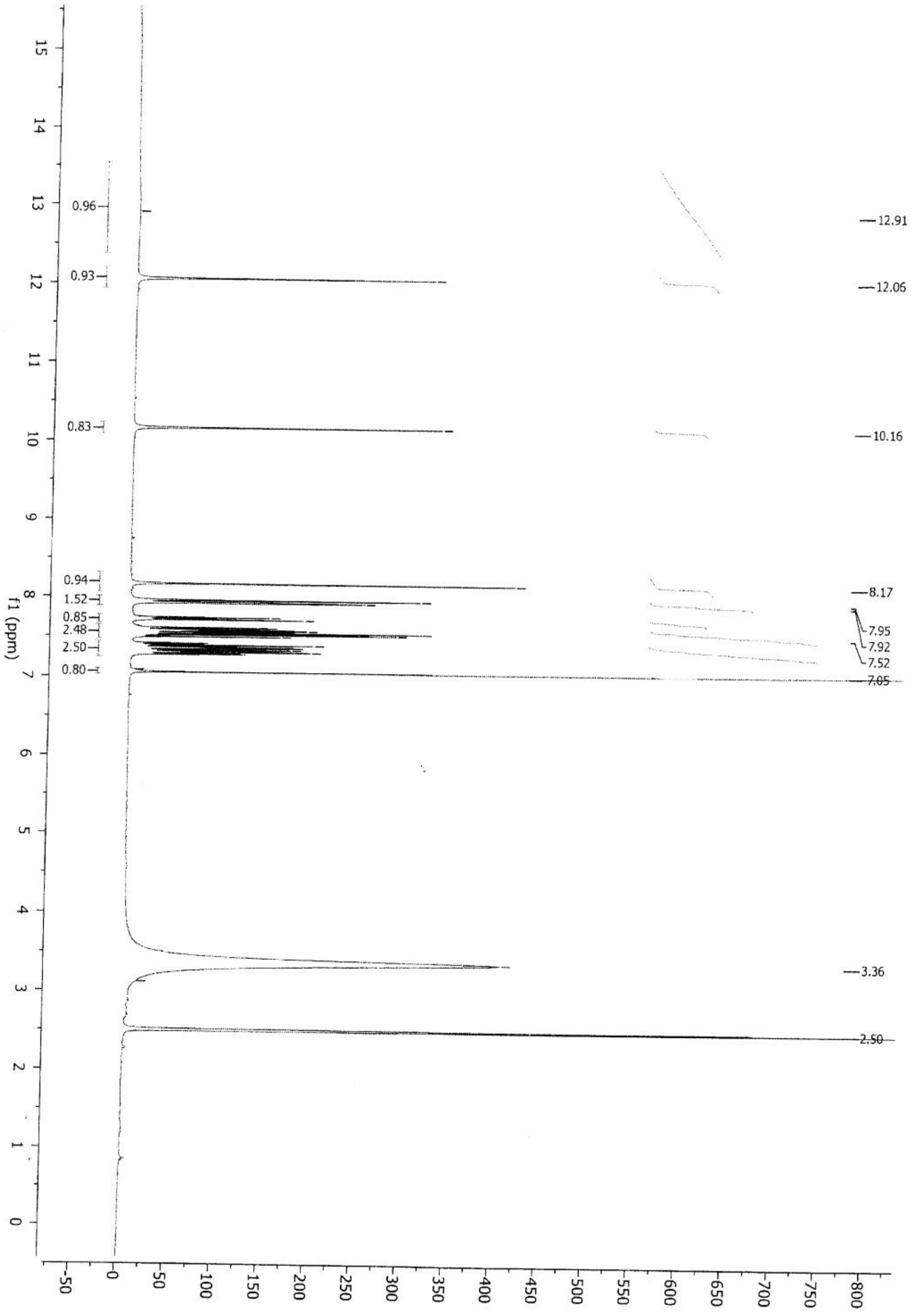

Figure S5. ^{1}H NMR spectra for **4**

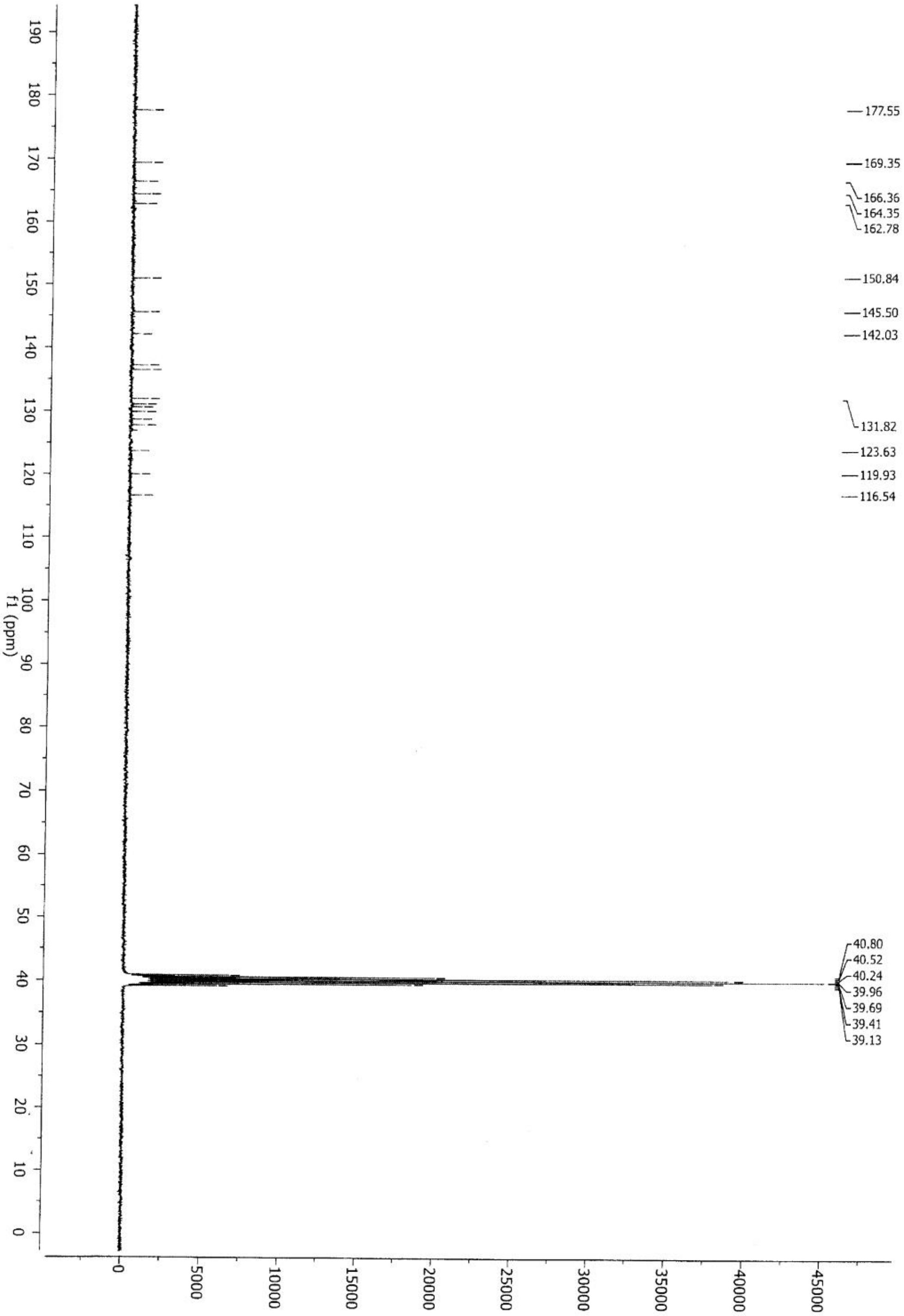

Figure S6. ^{13}C NMR spectra for **4**

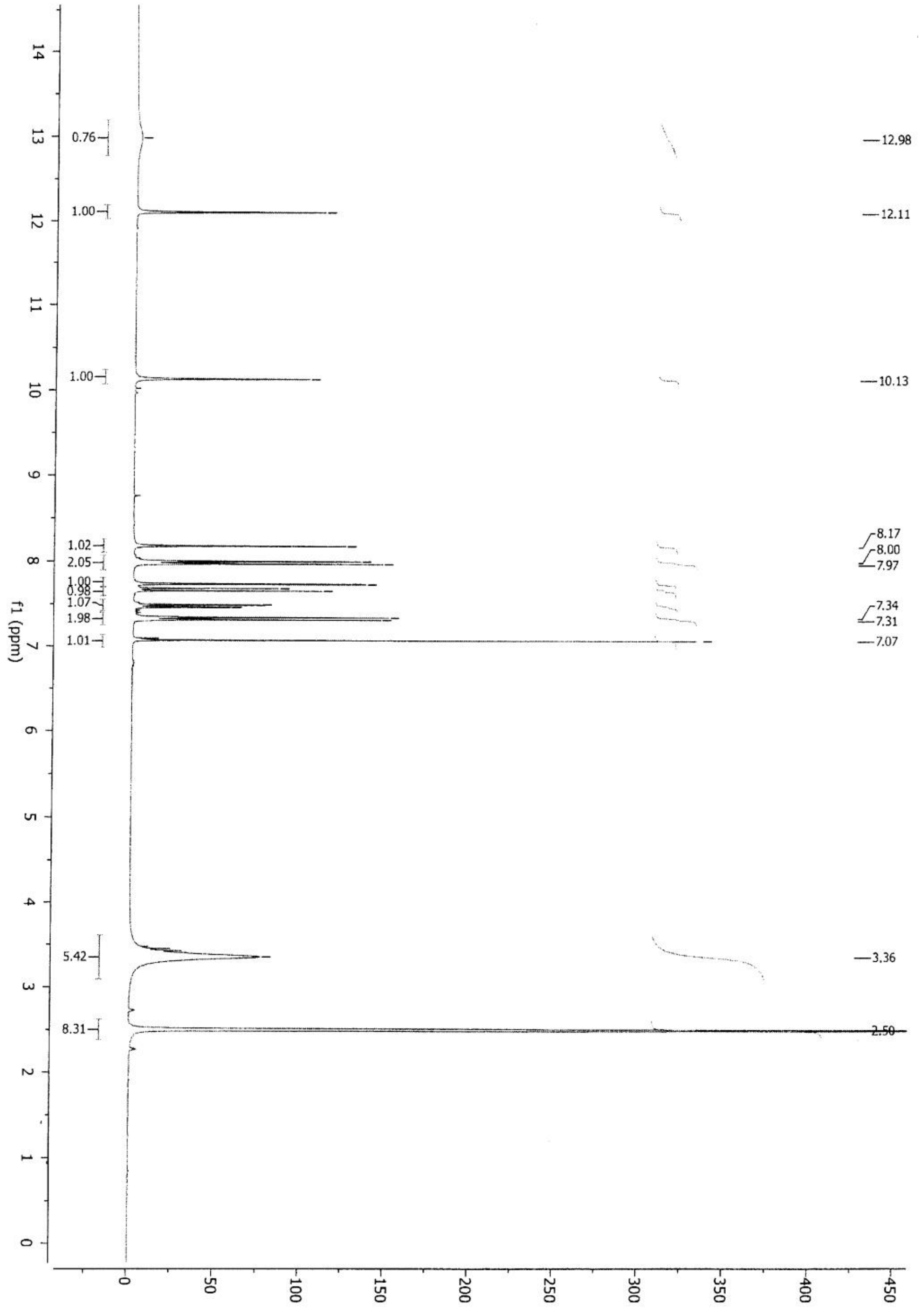

Figure S7. ^{1}H NMR spectra for **5**

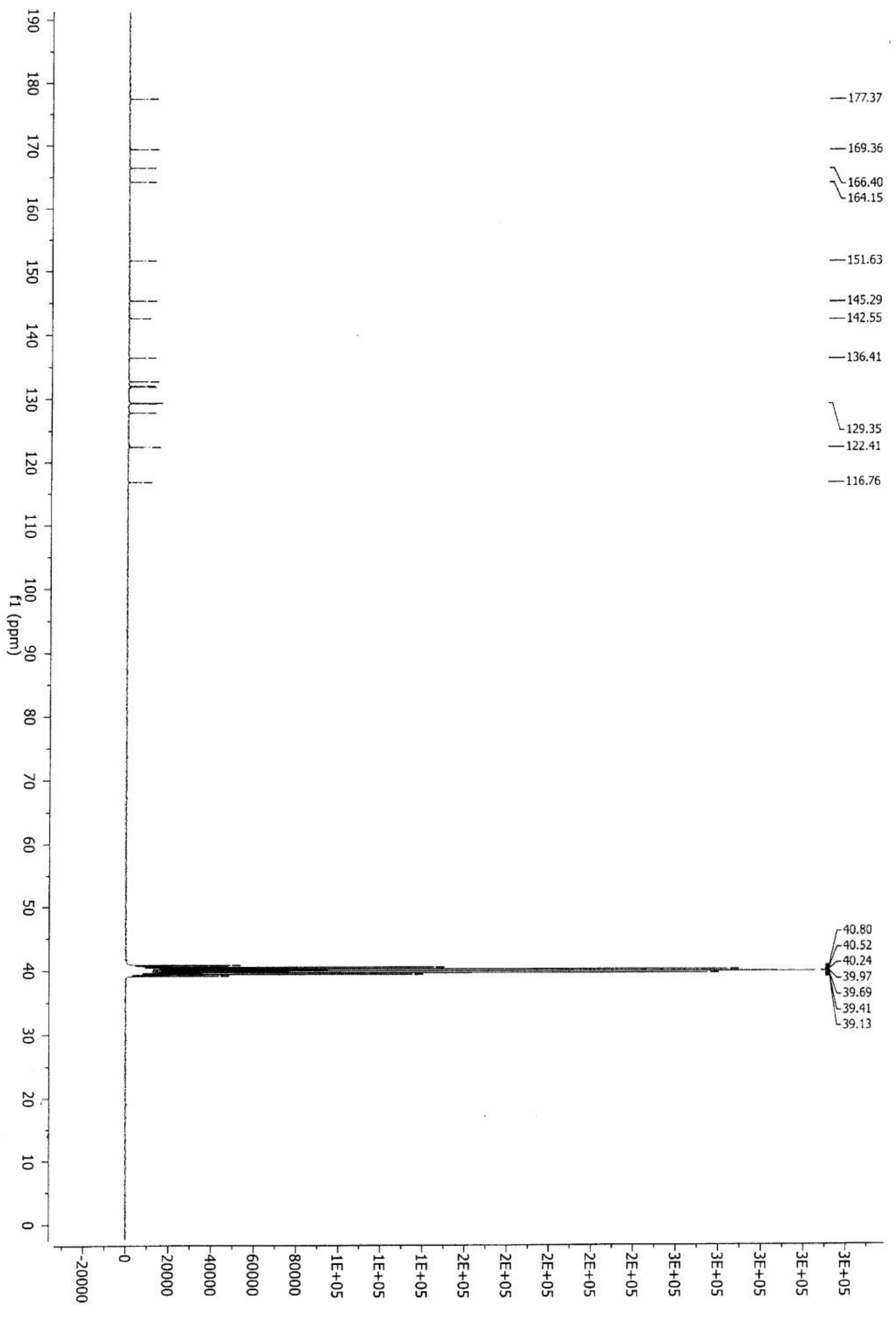

Figure S8. ^{13}C NMR spectra for **5**
